# Parsing Fabry Disease Metabolic Plasticity Using Metabolomics

**DOI:** 10.3390/jpm11090898

**Published:** 2021-09-08

**Authors:** Franklin Ducatez, Wladimir Mauhin, Agnès Boullier, Carine Pilon, Tony Pereira, Raphaël Aubert, Olivier Benveniste, Stéphane Marret, Olivier Lidove, Soumeya Bekri, Abdellah Tebani

**Affiliations:** 1 Department of Metabolic Biochemistry, Normandie University, UNIROUEN, INSERM U1245, CHU Rouen, 76000 Rouen, France; franklin.ducatez@gmail.com (F.D.); carine.pilon@chu-rouen.fr (C.P.); raphael.aubert@univ-rouen.fr (R.A.); soumeya.bekri@chu-rouen.fr (S.B.); 2 Department of Neonatal Pediatrics, Intensive Care, and Neuropediatrics, Normandie University, UNIROUEN, INSERM U1245, CHU Rouen, 76000 Rouen, France; stephane.marret@chu-rouen.fr; 3 Department of Internal Medicine, Groupe Hospitalier Diaconesses Croix Saint Simon, Site Avron & UMRS 974, 75013 Paris, France; wmauhin@hopital-dcss.org (W.M.); olidove@hopital-dcss.org (O.L.); 4 MP3CV-UR7517, CURS-Université de Picardie Jules Verne, Avenue de la Croix Jourdain, 80054 Amiens, France; Boullier.Agnes@chu-amiens.fr; 5 Laboratoire de Biochimie CHU Amiens-Picardie, Avenue de la Croix Jourdain, 80054 Amiens, France; 6 CHU Rouen, Institut de Biologie Clinique, 76000 Rouen, France; tony.pereira@chu-rouen.fr; 7 Department of Internal Medicine, Hôpital Pitié-Salpêtrière & INSERM U 974, 75013 Paris, France; olivier.benveniste@aphp.fr

**Keywords:** inborn errors of metabolism, Fabry disease, lysosomal storage diseases, metabolomics, systems biology, machine learning

## Abstract

Background: Fabry disease (FD) is an X-linked lysosomal disease due to a deficiency in the activity of the lysosomal α-galactosidase A (GalA), a key enzyme in the glycosphingolipid degradation pathway. FD is a complex disease with a poor genotype–phenotype correlation. FD could involve kidney, heart or central nervous system impairment that significantly decreases life expectancy. The advent of omics technologies offers the possibility of a global, integrated and systemic approach well-suited for the exploration of this complex disease. Materials and Methods: Sixty-six plasmas of FD patients from the French Fabry cohort (FFABRY) and 60 control plasmas were analyzed using liquid chromatography and mass spectrometry-based targeted metabolomics (188 metabolites) along with the determination of LysoGb3 concentration and GalA enzymatic activity. Conventional univariate analyses as well as systems biology and machine learning methods were used. Results: The analysis allowed for the identification of discriminating metabolic profiles that unambiguously separate FD patients from control subjects. The analysis identified 86 metabolites that are differentially expressed, including 62 Glycerophospholipids, 8 Acylcarnitines, 6 Sphingomyelins, 5 Aminoacids and 5 Biogenic Amines. Thirteen consensus metabolites were identified through network-based analysis, including 1 biogenic amine, 2 lysophosphatidylcholines and 10 glycerophospholipids. A predictive model using these metabolites showed an AUC-ROC of 0.992 (CI: 0.965–1.000). Conclusion: These results highlight deep metabolic remodeling in FD and confirm the potential of omics-based approaches in lysosomal diseases to reveal clinical and biological associations to generate pathophysiological hypotheses.

## 1. Introduction

Fabry disease (FD, OMIM #301500) is an X-linked inherited metabolic disease (IMD) due to lysosomal α-galactosidase A activity deficiency (GalA-EC 3.2.1.22), which has a key role in the glycosphingolipid degradation pathway, leading to cellular dysfunction and microvascular pathology [1]. The incidence ranges from 1 in 40,000 to 1 in 117,000 births in the general population [2]. However, this might be underestimated as some screening studies (Japan [3], Austria [4], northwestern Italy [5], United States (Missouri) [6] and Taiwan [7]) reported a higher incidence rate (1/1500–1/7000). The impairment of GalA generates a progressive accumulation of glycosphingolipid derivatives such as globotriaosylceramide (Gb3) and galabiosylceramide in the lysosome. This may occur in various cell types such as vascular, endothelial, renal, cardiac and nerve cells (neurons, Schwann cells) where the continuous deposition leads to serious cellular damage and organ failure [8]. Thus, damage to the kidney, heart and central nervous system will significantly decrease life expectancy [9]. Most Fabry patients show no symptoms in early life; however, symptoms may arise in childhood or adolescence. The classical phenotype in FD presents cornea verticillata, neuropathic pain, gastrointestinal dysfunction and angiokeratoma [10,11]. Serious complications are usually observed in adulthood and may include progressive renal insufficiency, cardiac complications (arrhythmia, hypertrophic cardiomyopathy) and/or cerebrovascular complications. Vascular ectasia and tortuosity could also be observed [10]. Pathogenic variants might present with low to absent residual GalA activity in males spanning the full disease clinical spectrum. In heterozygous females, the presentation is miscellaneous. This clinical variability might be due to the X-inactivation in female patients. Putative modifier genes could also explain part of the lack of *GLA* genotype–phenotype correlation. FD diagnosis is usually made by the deficiency in GalA activity in white blood cells from a blood sample [12], plasma/serum [13] or a dried blood spot [14]. Other samples could be used to identify a GalA activity deficiency such as lymphoblasts, cultured fibroblasts, tears or urine [15]. The diagnosis confirmation is done using molecular analysis of the *GLA* gene. While reliable for male patients, the enzymatic assessment is dubious for female carriers given the abovementioned random inactivation of the X-chromosome. In this case, molecular analysis is a very precious tool to detect heterozygous individuals. The storage product Gb3 concentrations are inconsistently increased in either plasma [16,17] or urine [18] in late onset forms and in female patients. For a greater discrimination, globotriaosylsphingosine (LysoGb3), a deacylated derivative of Gb3, is suggested. However false negatives in some female patients and in very late onset forms have been described [19,20]. Moreover, LysoGb3 does not correlate well with clinical events in patients under treatment, as recently illustrated in patients on migalastat-chaperone therapy [21]. Regarding treatment, Enzyme Replacement Therapy (ERT) using intravenous exogeneous human α-Galactosidase A has enhanced FD management. Currently, two ERTs are available: recombinant (Agalsidase β) [22] or gene-activated human α-Galactosidase A enzyme [23]. Substrate reduction therapy (SRT) is another approach aiming to reduce the synthesis of glucosylceramide via inhibition of the glucosylceramide synthase. Two molecules are currently under clinical trials: Venglustat and Lucerastat [24]. Recently, a new therapeutic strategy was also reported using a pharmacological chaperone that can help proper protein folding of the mutated protein to increase the enzymatic activity [25]. All of these therapeutic avenues aim to improve the quality of life of the patients and to slow the course of the disease [26]. An effective management of FD requires early diagnosis. This highlights the lack of robust surrogate markers and molecular understanding of FD pathogeny for effective diagnosis, patient stratification and personalized management [27,28]. Thus, a better understanding of FD biological plasticity might enhance our screening and diagnosis tools. 

The ongoing omics revolution has opened new avenues to interrogate biological systems through data-rich strategies at an unprecedented breadth, depth and scope in different fields, including IMD [29,30,31,32]. This omics surge is mainly driven by high throughput technologies, bioinformatics, data sciences and systems biology approaches. Such systems-based strategies promote unbiased, data-driven and hypothesis-free studies to explore health and disease states and get rid of hypothesis-driven aspects of conventional reductionist approaches [31]. In FD, several omics-based studies have been previously reported in FD [33,34,35,36,37,38,39,40,41,42,43,44]. We describe here a network-based targeted metabolomics study aiming to determine metabolic-based biological signatures that could discriminate Fabry patients from healthy subjects. In addition, we aimed to compare the unveiled metabolomic profile with routinely FD biomarkers.

## 2. Materials and Methods

### 2.1. Patients and Blood Samples

**Figure 1:**
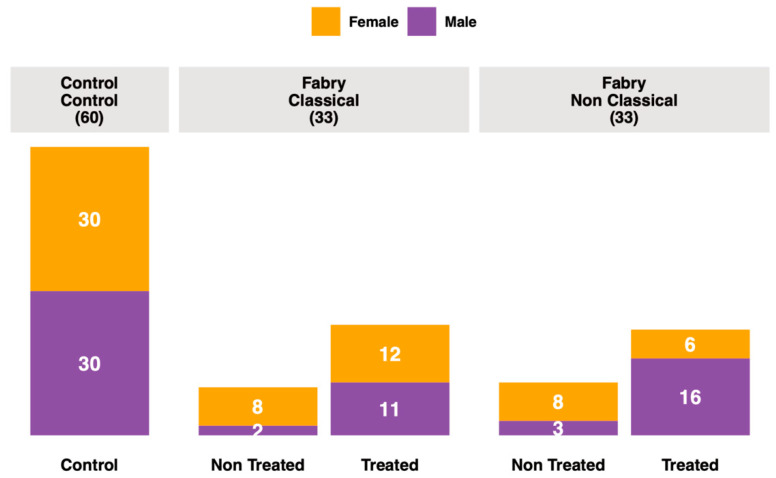
Cohort description.

Blood samples were retrieved from the French Fabry cohort (FFABRY), a French multicenter cohort of patients with an enzymatic and/or genetic diagnosis of FD [45]. A total of 66 patients were included: 33 with classical phenotype, including 20 females (age range: 25 to 75 years; mean age: 47 years) and 13 males (age range: 20 to 59 years; mean age: 38 years), and 33 with non-classic phenotype, including 14 females (age range: 17 to 66 years; mean age: 46 years) and 19 males (age range: 17 to 74 years; mean age: 49 years). A total of 45 were treated, 11 with Agalsidase α (8 classical and 3 non-classical), 21 with Agalsidase β (11 classical and 10 non-classical), 1 with Migalastat (non-classical), 10 with Agalsidase α and Agalsidase β (4 classical and 6 non-classical), 1 with Agalsidase α and Miglastat (non-classical), and 1 with all three, Agalsidase β, Agalsidase α and Migalastat (non-classical). No significant differences in age, sex or treatment between phenotype groups were observed. The mean cumulative treatment duration time was 6.4 years. Genotyping had been performed in 61 patients. For missense variants, 13 and 24 were found in classical and non-classical Fabry patients, respectively. There were 16 and 8 variants leading to a truncated protein (deletion, frameshift or non-sense mutations) in classical and non-classical Fabry patients, respectively. All these characteristics are presented in Supplementary Tables S1 and S2. Human control plasmas with no significant medical conditions were purchased from Biovit (West Sussex, UK). We analyzed plasma samples from 60 healthy donors, 30 males (age range: 20 to 55 years; mean age: 34 years) and 30 females (age range: 18 to 56 years; mean age: 37 years). The overall summary of the cohort is presented in Figure 1. 

The study was approved by the Institutional Ethics Committee Research (Ethics Board of Rouen University Hospital-CERNI E2016-21).

### 2.2. Targeted Metabolomics Analysis

All reagents required for the AbsoluteIDQ^®^ p180 analysis are included in the kit or provided by Biocrates Life Science AG (Innsbruck, Austria). Sample preparation was carried out according to the manufacturer’s protocol [46,47]. Briefly, 10 µL of plasma was transferred to the upper 96-well plate and dried under a nitrogen stream. Thereafter, 50 µL of a 5% PITC solution was added to derivatize amino acids and biogenic amines. After incubation, the spots were dried again before the metabolites were extracted using 5 mM ammonium acetate in methanol (300 µL) into the lower 96-well plate for analysis after further dilution using the MS running solvent A. Quantification was carried out using isotopically labeled internal standards and a calibration curve [46,47]. The full list of 188 measured metabolites is presented Supplementary Table S3. The AbsoluteIDQ^®^ p180 kit is a fully automated assay based on phenylisothiocyanate (PITC) derivatization of the target analytes in bodily fluids using internal standards for quantitation. Amino acids and biogenic amines are determined in LC-MS mode, acylcarnitines, phospholipids, sphingomyelins, and the sum of hexoses are analyzed in flow injection analysis (FIA). The analyses were performed following the instructions of the kit manufacturer: autosampler temperature at 10 °C, injection volume at 10 µL, reversed-phase HPLC gradient using HPLC grade water and acetonitrile, both with 0.2% formic acid (FA), flow rate at 0.5 mL/min. For the FIA, only acetonitrile with 0.2% FA was used with a max flow rate of 0.2 mL/min. Liquid chromatography instrument prominence Shimadzu UFLC System (Shimadzu, Prominence, Kyoto, Japan) was used coupled to the 4000 Qtrap mass spectrometer (Sciex, Framingham, MA, USA) with an electrospray ion source. Data acquisition and processing were performed using the Analyst 1.5 software (Sciex, Framingham, MA, USA).

### 2.3. Plasma LysoGb3 Analysis

The LysoGb3 concentration was measured as previously described [45]. Using plasma samples and ultra-performance liquid chromatography coupled to tandem mass spectrometry (UPLC-MS/MS). In glass tubes, EDTA-plasma was mixed with glycine-LysoGb3 (100 ng/mL) as an internal standard. Proteins were precipitated with methanol:acetone 1:1 (*v*/*v*), sonicated for 30 s in a bath sonifier and vortexed. After centrifugation, 8 min at 16,000× *g*, the supernatant was transferred into new tubes and dried. For UPLC-LCMS/MS analysis, the residue was redissolved in methanol. Quantitative analysis of LysoGb3 was performed on a TQD mass spectrometer coupled to an Acquity UPLC system (Waters^®^) and equipped with an Acquity BEH-C18 column. Elution was achieved by mobile phase A, consisting of 37% methanol, 63% water containing 1 mM ammonium formiate and 0.1% formic acid, and mobile phase B, consisting of 100% methanol containing 1 mM ammonium formiate and 0.1% formic acid. A calibration curve was generated by a serial dilution of LysoGb3 (Matreya-LLC) in methanol, with concentrations ranging from 100 to 1.56 ng/mL.

### 2.4. Alpha-D-Galactopyranosidase Activity Analysis

Alpha-D-galactopyranosidase enzymatic activity was assessed in isolated blood leukocytes using a fluorometric assay [13]. Hexosaminidase activity was also determined as an enzyme control to confirm leukocyte integrity. The residual enzymatic activity (REA) is defined as the ratio of enzyme activity measured in a sample to the activity measured with a control.

### 2.5. Data Analysis

Data matrix was log-transformed and pareto-scaled [48]. Missing values were imputed using nearest neighbor averaging algorithm using the impute.knn function in the impute R package. Univariate analyses were performed using *t*-tests to identify discriminatory features between the assessed groups. Limma package [49] was used for differential analysis with sex and age taken into account by adding it as covariate. Spearman correlation analysis was performed using R software. Euclidean distance was used as a similarity measure in the clustering analysis. Principal Component Analysis was used as dimension reduction technique using log-transformed and pareto-scaled dataset. False discovery rates were corrected using the Benjamini–Hochberg–Yekutieli method [50], and *p* < 0.05 was considered statistically significant. For network analysis, the first step was to compute several partial correlation matrices (PCM) [51]. Three kinds of PCMs were calculated: control + disease samples, control samples only, and all the combinations of samples including control + “disease-minus-one-patient” to get patient specific networks. Networks were then constructed from each PCMs data matrix and pruned with each other to get specific networks. The idea of network pruning is to remove edges in a general network that are also found in a more specific network. So, we pruned the “disease + control” network with the “control” network in order to keep only edges that are disease specific. Thus, this step results in a “disease-specific” network. This step has been done using the CTD R package [52]. Using the same strategy, networks of controls + “disease-minus-one” samples were pruned with the control-samples network to obtain a “disease-specific-minus-one-patient” network. This network was then pruned with the “disease-specific” network calculated above in order to extract “patient-specific” metabolic signatures. A summary overview of the network strategy is presented in Supplementary Figure S1. The metabolites present in all of these “patient-specific” networks were selected to build a Consensus Network and enable the visualization of key metabolic signatures for the disease. To test the discriminatory power of this signature, Random Forest models were tuned for every possible combination of metabolites from the Consensus Network. Random Forest models were built using the ranger package [53] and the caret package in R [54]. The models were tuned over ~50 repeats to obtain robust classification probabilities. Performances of the models were assessed with the MLeval package in R. The main metric for predictive performance assessment was the area under the curve (AUC) for the resulting receiver operating characteristic (ROC) curve. All data analyses and visualizations were performed using R software [55].

## 3. Results

**Figure 2:**
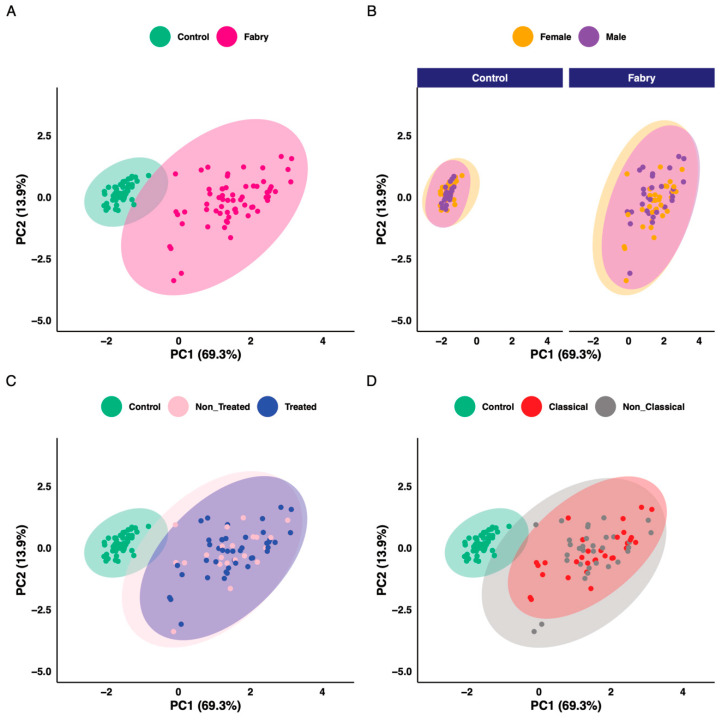
Principal component analysis scores plot. (**A**) Colored according to disease status. (**B**) Colored according to sex. (**C**) Colored according to treatment status. (**D**) Colored according to phenotype status.

**Figure 3:**
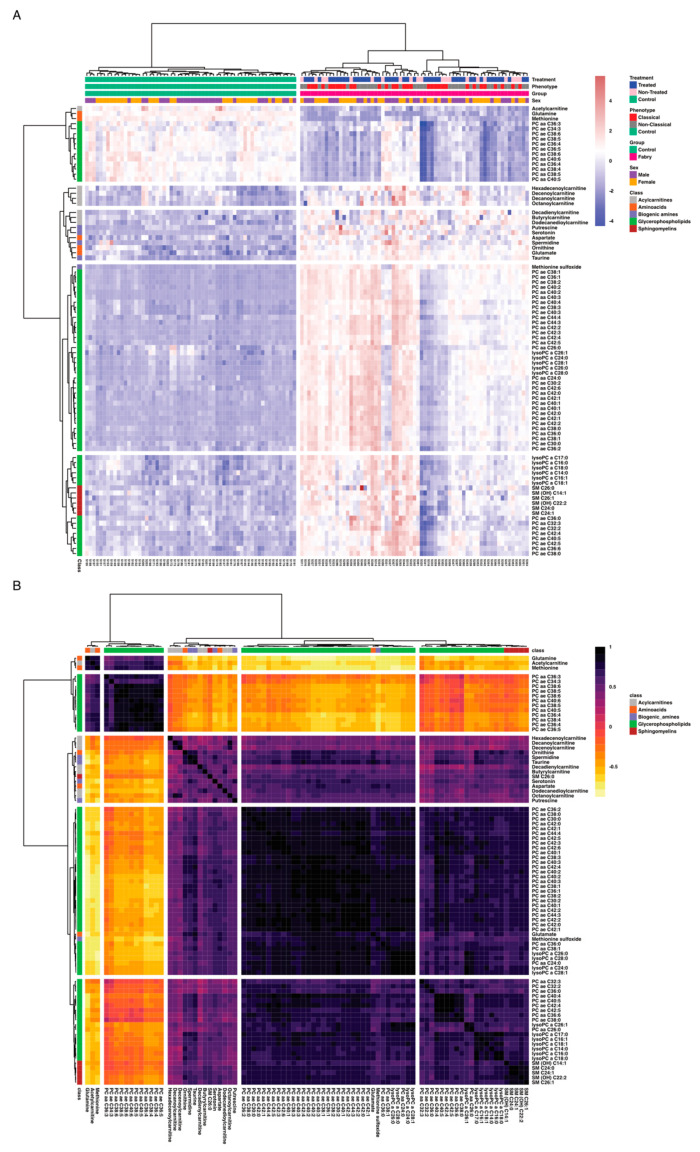
(**A**) Sample similarity heatmap. The colors refer to concentration z-scores. (**B**) Metabolites correlation heatmap. The color refers to Spearman correlation coefficients.

**Figure 4:**
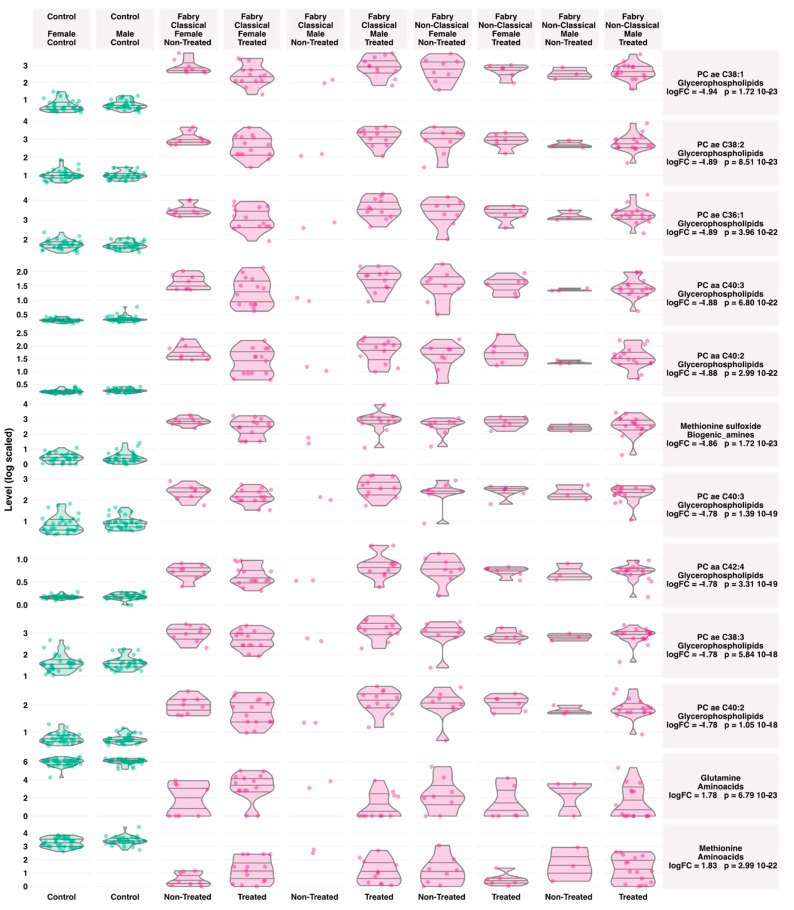
Boxplots of the top varying metabolites between Fabry and control samples. Lines in the violin plots refer to quantiles (0.25, 050 and 0.75).

The aim of this work is to explore metabolic profile differences between Fabry and control samples using plasma-targeted metabolomics. The full data matrix with samples characteristics is presented in Supplementary Table S2. To analyze the data, the first approach was to use an unsupervised analysis to track samples’ clustering trends based on the underlying metabolic profiles. The principal component analysis score plot revealed a clear separation between Fabry and control samples (Figure 2A). This separation was mainly observed on the PC1 dimension which explains alone 69.3% of the variance of the dataset. No sex, treatment or disease phenotype separation were observed on the PCA (Figure 2B–D). The PCA scores’ matrices are presented in Supplementary Table S4. To go further, we performed a differential analysis between the two groups Fabry versus control samples. The analysis identified 86 metabolites that are differentially expressed. The full list of metabolites and their related statistics are presented in Supplementary Table S5. The metabolites include 62 Glycerophospholipids, 8 Acylcarnitines, 6 Sphingomyelins, 5 Aminoacids and 5 Biogenic Amines. To visualize the discriminant effect of these metabolites on the samples, we present in Figure 3A a heatmap of the correlation between metabolites (rows) and samples (columns). The full correlation matrix is presented in Supplementary Table S6. The heatmap clearly shows two main clusters belonging to Fabry and Control samples. This clustering is driven by the respective metabolic profile in each sample. Thus, we have performed correlation analysis between the differentially expressed metabolites. The results are presented in a heatmap (Figure 3B). The figure shows four main clusters with high intraclass correlation, especially between glycerophospholipids and sphingomyelins, Acylcarnitines and aminoacids. The top 12 differentially expressed metabolites are presented in boxplots (Figure 4). These include 3 amino acids: Glutamine (logFC = 1.78, *p*-value = 6.79 × 10^−23^), Methionine (logFC = 1.83, *p*-value = 2.99 × 10^−22^), Methionine sulfoxide (logFC = −1.86, *p*-value = 1.72 × 10^−23^), and 9 phosphatidylcholins: PC ae C38:1 (logFC = −1.94, *p*-value = 1.72 × 10^−23^), PC ae C38:2 (logFC = −1.89, *p*-value = 8.51 × 10^−23^), PC aa C40:2 (logFC = −1.88, *p*-value = 2.99 × 10^−22^), PC ae C36:1 (logFC = −1.89, *p*-value = 3.96 × 10^−22^), PC aa C40:3 (logFC = −1.88, *p*-value = 6.8 × 10^−22^), PC ae C40:3 (logFC = −1.78, *p*-value = 1.39 × 10^−19^), PC aa C42:4 (logFC = −1.78, *p*-value = 3.31 × 10^−19^), PC ae C40:2 (logFC = −1.78, *p*-value = 1.05 × 10^−18^), PC ae C38:3 (logFC = −1.78, *p*-value = 5.84 × 10^−18^).

**Figure 5:**
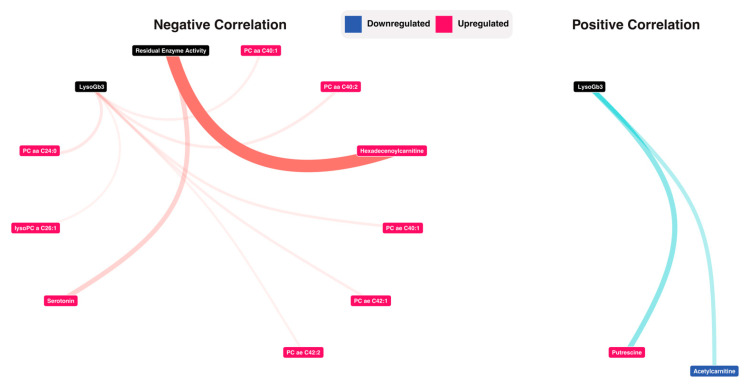
Correlation analysis between LysoGb3, Residual enzyme activity and the metabolomic data. Detailed results are presented in supplementary Table S7.

To investigate the correlation between the associations between the retrieved metabolic profile and residual enzyme activity and LysoGb3, we used Spearman correlations. For LysoGb3, the analysis yielded nine negative correlations (PC aa C24:0 vs. LysoGb3: rho = −0.3 adjusted *p*-value = 3.10 × 10^−2^, PC aa C40:2 vs LysoGb3: rho = −0.3 adjusted *p*-value = 3.41 × 10^−2^, PC ae C40:1 vs. LysoGb3: rho = −0.29 adjusted *p*-value = 3.60 × 10^−2^, PC ae C42:1 vs. LysoGb3: rho = −0.29 adjusted *p*-value = 3.76 × 10^−2^, PC aa C40:1 vs. LysoGb3: rho = −0.29 adjusted *p*-value = 3.92 × 10^−2^, PC ae C42:2 vs. LysoGb3: rho = −0.28 adjusted *p*-value = 4.50 × 10^−2^, lysoPC a C26:1 vs LysoGb3: rho = −0.28 adjusted *p*-value = 4.60 × 10−^2^), and two positive correlations (Acetylcarnitine vs. LysoGb3: rho = 0.33 adjusted *p*-value = 1.83 × 10^−2^, Putrescine vs. LysoGb3: rho = 0.35 adjusted *p*-value = 1.05 × 10^−2^). For residual enzyme activity, two negative correlations were observed (Hexadecenoylcarnitine vs. Residual Enzyme Activity: rho = −0.48 adjusted *p*-value = 4.87 × 10^−4^, Serotonin vs. Residual Enzyme Activity: rho = −0.34 adjusted *p*-value = 1.85 × 10^−2^). A network visualization is presented in Figure 5, and full results are presented in Supplementary Table S7. 

**Figure 6:**
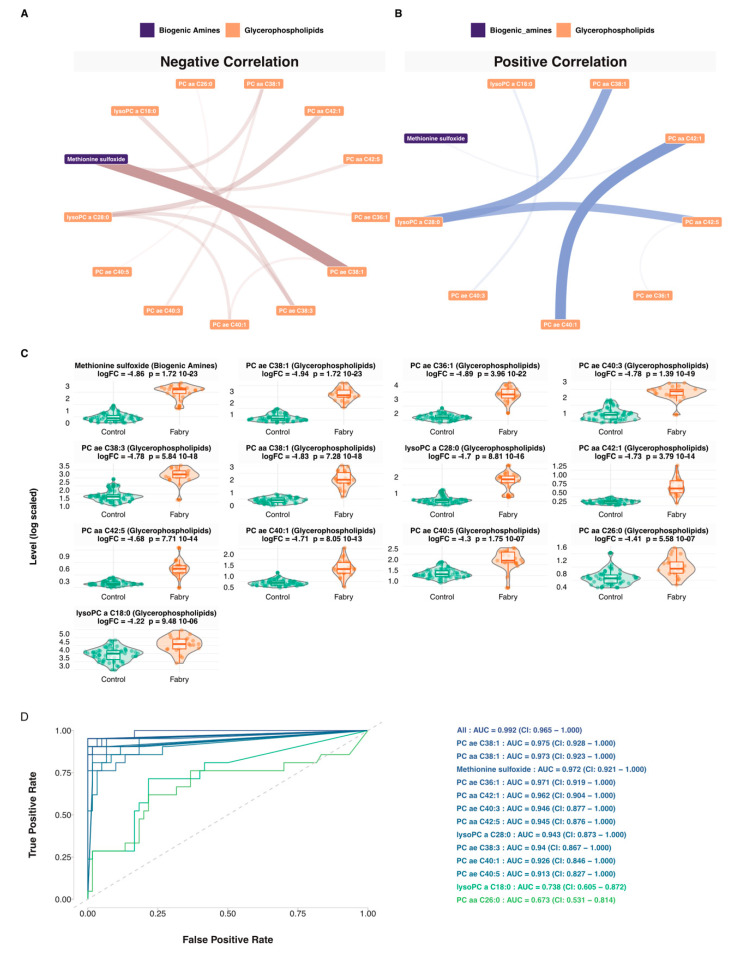
Network and machine learning analysis. (**A**) Negative partial correlation network visualization of the Fabry consensus plasma metabolic signature. The size of the ribbon is proportional to the correlation. (**B**) Positive partial correlation network visualization of the Fabry consensus plasma metabolic signature. The size of the ribbon is proportional to the correlation. (**C**) Boxplots of the consensus plasma metabolic signature. (**D**) ROC curves of the Random Forest predictive models, including 1 biogenic amine (Methionine sulfoxide), 2 lysophosphatidylcholines (lysoPC a C18:0, lysoPC a C28:0) and 10 glycerophospholipids (PC ae C38:1, PC aa C38:1, PC ae C36:1, PC aa C42:1, PC ae C40:3, PC aa C42:5, PC ae C38:3, PC ae C40:1, PC ae C40:5, PC aa C26:0).

For a more personalized assessment of the results at the patient’s level, a network-based strategy was used. This was based on generating different networks using control, disease or both samples. Using these networks, 21 Fabry-specific metabolic signatures were extracted that are presented in Supplementary Figures S1–S21. Networks and the full list for each sample are presented in Supplementary Tables S8–S11. Then, to build a consensus network, we identified the most redundant metabolites found in all of the patients’ signatures. The consensus network included 13 metabolites: 1 biogenic amine (Methionine sulfoxide), 2 lysophosphatidylcholines (lysoPC a C18:0, lysoPC a C28:0) and 10 glycerophospholipids (PC ae C38:1, PC aa C38:1, PC ae C36:1, PC aa C42:1, PC ae C40:3, PC aa C42:5, PC ae C38:3, PC ae C40:1, PC ae C40:5, PC aa C26:0). Correlation network visualizations are presented in Figure 6A,B. The full results are presented in Supplementary Table S12. To have an overview of the expression levels of these metabolites, boxplots are shown in Figure 6C. Based on this consensus signature, we explored the predictive performance of each of the 13 metabolites and all their possible combinations using predictive Random Forest models. Area under curve and ROC curves were used as performance metrics. All model-related results are presented in Supplementary Table S13. The 13 univariate models and their combination are shown in Figure 6 These models showed the following predictive performances: PC ae C38:1-AUC = 0.975 (CI: 0.928–1.000), PC aa C38:1-AUC = 0.973 (CI: 0.923–1.000), Methionine sulfoxide-AUC = 0.972 (CI: 0.921–1.000), PC ae C36:1-AUC = 0.971 (CI: 0.919–1.000), PC aa C42:1-AUC = 0.962 (CI: 0.904–1.000), PC ae C40:3-AUC = 0.946 (CI: 0.877–1.000), PC aa C42:5-AUC = 0.945 (CI: 0.876–1.000), lysoPC a C28:0-AUC = 0.943 (CI: 0.873–1.000), PC ae C38:3-AUC = 0.94 (CI: 0.867–1.000), PC ae C40:1-AUC = 0.926 (CI: 0.846–1.000), PC ae C40:5-AUC = 0.913 (CI: 0.827–1.000), lysoPC a C18:0-AUC = 0.738 (CI: 0.605–0.872), PC aa C26:0-AUC = 0.673 (CI: 0.531–0.814). Overall, most of the models showed an AUC higher than 0.90. It is worth mentioning that one of the most predictive model includes all the metabolites and showed an AUC = 0.992 (CI: 0.965–1.000). 

## 4. Discussion

Fabry disease is an IMD that displays a clinical heterogeneity. Currently, LysoGb3 is the most reliable diagnostic biomarker for Fabry disease. However, it fails in diagnosing some non-classical phenotypes or female patients. Since an early diagnosis of this rare disease allows the optimization of patient management, it is essential to uncover disease-specific biomarkers that could allow the stratification of patients and provide tools for treatment follow-up. To achieve this goal, we conducted a targeted metabolomic study in a series of Fabry patients and control individuals. A total of 188 metabolites, including acylcarnitines, biogenic amines, amino acids, glycerophospholipids and sphingomyelins, were quantified in plasma samples. Unsupervised multivariate analysis of the concentrations of these metabolites showed a very clear discrimination of metabolomic profiles between the control and Fabry groups. However, we found no specific metabolic patterns related to gender, disease phenotype (classical vs. non-classical) or disease treatment. Furthermore, this targeted metabolomic analysis shows that 86 metabolites have differential expression between Fabry and control samples. The main unveiled biochemical classes include glycerophospholipids, acylcarnitines, amino acids, biogenic amines and sphingomyelins. To parse the metabolic complexity of these patterns, we used an integrative network-based strategy coupled to a machine learning approach that uncovered a consensual biosignature that is specifically increased in Fabry samples compared to controls. It includes 1 biogenic amine (Methionine sulfoxide), 2 lysophosphatidylcholines (lysoPC a C18:0, lysoPC a C28:0) and 10 glycerophospholipids (PC ae C38:1, PC aa C38:1, PC ae C36:1, PC aa C42:1, PC ae C40:3, PC aa C42:5, PC ae C38:3, PC ae C40:1, PC ae C40:5, PC aa C26:0).

These results show alterations in the metabolism of sphingomyelins and glycerophospholipids. Among the 15 metabolites most significantly altered between controls and Fabry patients, the majority belong to the class of glycerophospholipids (GPL), the components of which are important parts of cell membrane and are also involved in many biological processes such as inflammation and cell differentiation [56]. For example, the length of the fatty acid chain of which they are composed and their degree of saturation have an impact on membrane fluidity and permeability. GPL also serve as reservoirs for second messengers that will be released under the action of phospholipases. Zhang et al. showed that lipid metabolism can contribute to the pro- or anti-inflammatory activities of macrophages by modulating, for example, the fluidity of the membrane and are thus biomarkers of the activation state of macrophages [57]. Depending on the activation stimulus, activated macrophages are divided into 2 main groups: M1 macrophages (pro-inflammatory phenotype) and M2 macrophages (anti-inflammatory phenotype). This activation leads to a modification of the GPL from saturated to polyunsaturated GPLs. In addition, M1 has more GPLs than lysophospholipids, unlike M2. Since Fabry disease is an inflammatory disease, it is therefore interesting to note that most of the GPLs significantly modified in our metabolomic study are GPLs containing polyunsaturated fatty acids. GPLs are also found largely in neural cell membranes, and studies have shown that abnormal metabolism of GPL is associated with neuroinflammation and neurodegeneration [56]. For example, alterations in the GPL composition of the neural membrane have been shown to occur in neurological pathologies such as Alzheimer’s disease [58] and Parkinson’s disease [59]. Of note, GPL are also components of lipoproteins, particularly low-density lipoproteins (LDL), and it is now accepted that polyunsaturated fatty acids are more oxidation-sensitive [60]. The oxidation of LDL is a key step in the development of atherosclerosis [61]. Thus, the increase in polyunsaturated GPL in Fabry disease may therefore play a role in the occurrence of cardiovascular events in Fabry patients with increased oxidative stress.

Another metabolite of potential interest for Fabry disease is methionine, which is significantly decreased in Fabry patients compared to controls. Indeed, methionine is an essential amino acid that not only plays a proteinogenic role, but also intervenes in several important metabolic pathways such as the cycle of re-methylation of homocysteine to methionine in the presence of folates. This result is suggestive of an alteration in the homocysteine re-methylation cycle. Studies seem to indicate that Fabry patients have increased homocysteine concentrations even in the absence of chronic renal failure or vitamin deficiency [62,63,64]. However, the exact mechanism of this increase is not yet clearly understood. Of note, renal insufficiency may be associated with increased plasmatic levels of S-adenosylmethionine (SAM) while folate and methionine levels remain normal [65]. Studies have also shown that methylated forms of Gb3 are present in the plasma and urine of Fabry patients [66]. This might involve the metabolism of SAM, which is derived from methionine. SAM is a methyl donor and a regulator of epigenetics. The role of epigenetics, including DNA methylation, has already been suggested in lysosomal storage diseases [67] and might be involved in the lack of genotype–phenotype correlation in Fabry disease. Patients with Fabry disease also present autophagy impairments [68]. Yanagisawa et al. showed that deregulation of DNA methylation on the *GLA* gene is associated with this dysfunction and that there is a correlation between symptom severity, autophagy dysfunction and methylation of the mutant allele [69].

Interestingly, our analysis clearly shows that not only methionine level is decreased, but its oxidized form, methionine sulfoxide (Met-SO), is increased in patients compared to controls. One of the peculiarities of methionine is that it is very susceptible to oxidation, due to its cysteine sulfide groups. The oxidation of methionine modifies the physicochemical properties of proteins and consequently modulates their function. Numerous studies have shown an increase in oxidative stress in Fabry disease [70,71,72,73,74,75]. In particular, it has been shown that the accumulation of Gb3 induces oxidative stress [71,73,74,75] and that there is a correlation between Gb3 and oxidative stress [76]. Biancini et al. also reported an increase in lipid and protein oxidation and inflammation in Fabry patients with decreased antioxidant defenses [71] such as heme oxygenase 1 (HO1) [74]. Fabry patients have endothelial dysfunction [63,77,78]. Namdar et al. showed that the endothelial dysfunction is due to Gb3 accumulation [79]. This is partly due to a decrease in nitric oxide (NO) production by the enzyme endothelial NO synthase (eNOS). Indeed, under physiological conditions, eNOS is associated with the cofactor BH4 to produce NO. In a context of increased oxidative stress, BH4 is oxidized to BH2, and the eNOS thus decoupled from its cofactor no longer produces NO, but superoxide anion, a radical oxygen species (ROS), thus increasing oxidative stress. Shen’s team showed that BH4 was decreased in patients with Fabry disease [80]. Moreover, a decrease in superoxide dismutase 2, the mitochondrial enzyme responsible for the degradation of the superoxide anion, was observed in these patients [81]. The oxidation of methionine to methionine sulfoxide (Met-SO) is a mechanism by which proteins protect themselves from oxidative stress, and to protect the cell from radical oxygen species (ROS), the methionine SO reductase system intervenes to subsequently reduce Met-SO to methionine [82]. Therefore, Met-SO is considered a marker of the systemic oxidative state of the organism [83,84].

One of the limitations of our study is related to the small number of patients studied. Thus, our results will need to be confirmed in a larger cohort to verify the predictive nature of these metabolites in Fabry disease and to be able to use them as diagnostic and treatment monitoring tools, particularly in heterozygous women and moderate forms of the disease. It would also be promising to adjust these metabolites to clinical manifestations and assess their predictive performances of disease progression. 

## 5. Conclusions

This metabolomic study allowed us to unveil specific metabolic patterns in Fabry disease. The identification of specific pathological biosignatures provides a better understanding of the disease and in particular the important role of glycerophospholipids and oxidative stress in its pathophysiology. Consideration should be given to combining ERT or SRT treatments with an oxidative stress inhibitor treatment and using Met-SO as a biomarker for treatment monitoring. Moreover, this highlights the potential of using integrative omics and systems-based techniques to parse the genotype–phenotype complexity of FD. 

## Data Availability

All the data that support the findings are presented in the manuscript and the supplementary material.

## Supplementary Materials

The following are available online at https://www.mdpi.com/article/10.3390/jpm11090898/s1, Table S1: Cohorte Overview; Table S2: Metabolomics Data Matrix; Table S3: Variable metadata; Table S4: Principal Component Analysis Scores; Table S5: Differential Expression Analysis; Table S6: Metabolite Spearman Correlation Matrix; Table S7: Spearman Correlation with LysoGb3 and Residual Enzymatic Activity; Table S8: Partial Correlation Matrix including all samples; Table S9: Partial Correlation Matrix including only control samples; Table S10: Pruned network using all samples network; Table S11: Patient-specific pruned networks; Table S12: Consensus network; Table S13: All Random Forest Models.
