# Supplementary material for: Parsing Fabry Disease Metabolic Plasticity Using Metabolomics
Source: J Pers Med. 2021 Sep 8;11(9):898. doi: 10.3390/jpm11090898 (PMC8468728; doi:10.3390/jpm11090898)
Supplement: Supplementary file 1 [file jpm-11-00898-s001.zip › Ducatez_et_al_supplmentary_fabomics_20210731.pdf]

Parsing Fabry Disease Metabolic Plasticity using Metabolomics

Franklin Ducatez [1, 2], Wladimir Mauhin [3], Agnès Boullier [4, 5], Carine Pilon [1], Tony Pereira [6], Raphaël Aubert [1], Olivier Benveniste [7], Stéphane Marret [2], Olivier Lidove [3], Soumeya Bekri [1], Abdellah Tebani [1]*

[1] Normandie Univ, UNIROUEN, INSERM U1245, CHU Rouen, Department of Metabolic Biochemistry, 76000 Rouen, France; abdellah.tebani@chu-rouen.fr (A.T.); carine.pilon@chu-rouen.fr (C.P.); franklin.ducatez@gmail.com (F.D.); raphael.aubert@univ-rouen.fr (R.A.); soumeya.bekri@chu-rouen.fr (S.B.)

[2] Normandie Univ, UNIROUEN, INSERM U1245, CHU Rouen, Department of Neonatal Pediatrics, Intensive Care, and Neuropediatrics, 76000 Rouen, France; stephane.marret@chu-rouen.fr (S.M.); franklin.ducatez@gmail.com (F.D.)

[3] Department of Internal Medicine, Groupe Hospitalier Diaconesses Croix Saint Simon, Site Avron, 75020 Paris & UMRS 974 75013 Paris, France; wmauhin@hopital-dcss.org (W.M.); olidove@hopital-dcss.org (O.L.)

[4] MP3CV-UR7517, CURS-Université de Picardie Jules Verne, Avenue de la Croix Jourdain, F-80054 Amiens, France ; Boullier.Agnes@chu-amiens.fr (A.B.)

[5] Laboratoire de Biochimie CHU Amiens-Picardie, Avenue de la Croix Jourdain, F-80054 Amiens, France ; Boullier.Agnes@chu-amiens.fr (A.B.)

[6] CHU Rouen, Institut de Biologie Clinique, 76000 Rouen, France; tony.pereira@chu-rouen.fr (T.P.)

[7] Department of Internal Medicine, Hôpital Pitié-Salpêtrière, Paris, France & INSERM U 974, 75013 Paris, France ; olivier.benveniste@aphp.fr (O.B.)

* Correspondence:

Dr. Abdellah Tebani

Department of Metabolic Biochemistry

Rouen University Hospital,

37 Boulevard Gambetta

76000 Rouen Cedex, France.

abdellah.tebani@chu-rouen.fr

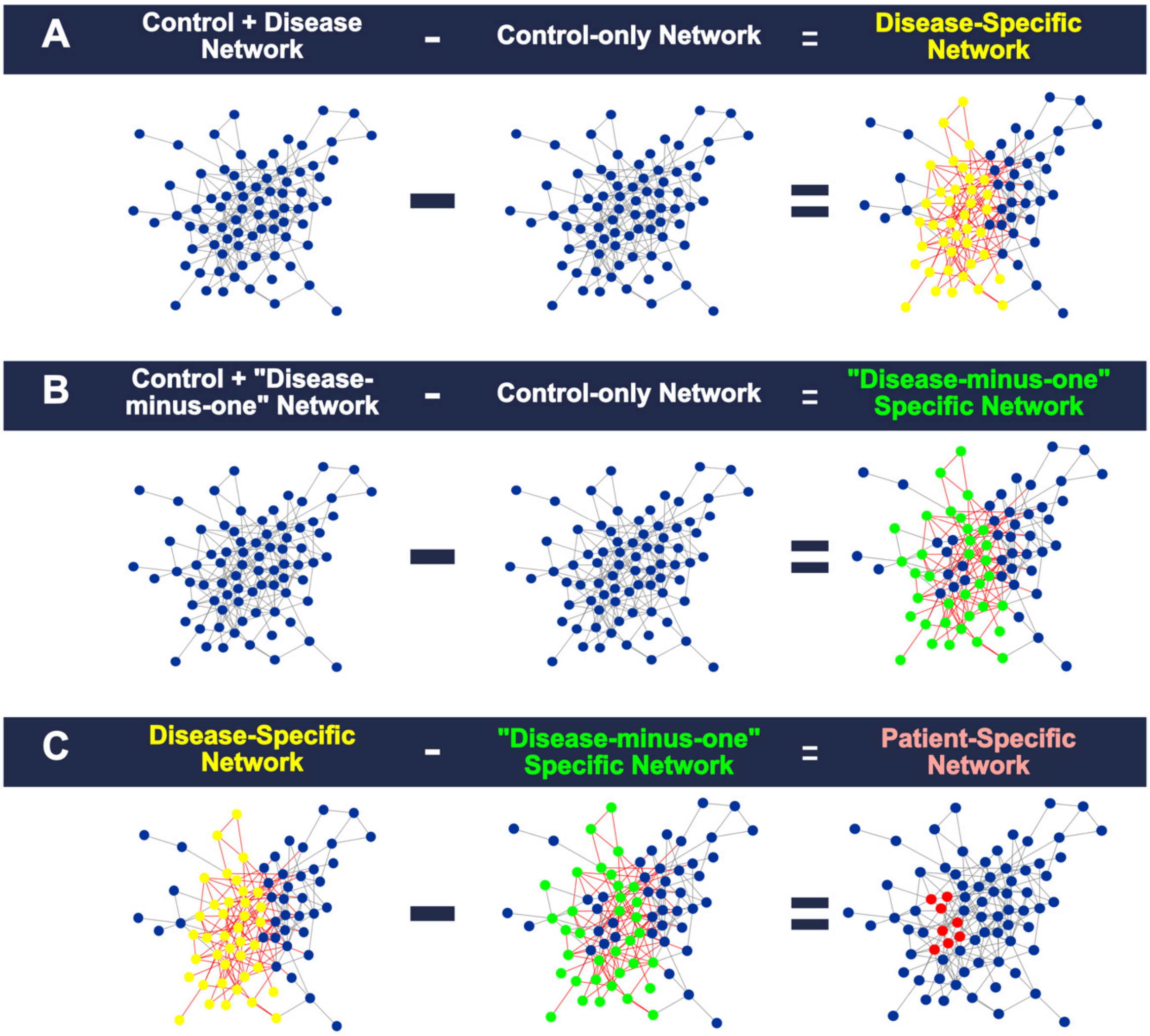

Supplementary Figure S1. Network analysis overview. Networks are built using Partial Correlation. The first network ("disease-control" network) is learned from disease profiles and control profiles. A second network composed of only control ("control-only" network). A third network is composed of control profiles and disease excluding one patient at a time ("Disease-minus-one" network). This third network is built iteratively to cover all the patients. A final pruning stage subtracts edges from the disease-control network that are also found either in A) the control-only network, outputting the disease-specific network B) "Disease-minus-one" specific network or C) Patient-specific network.

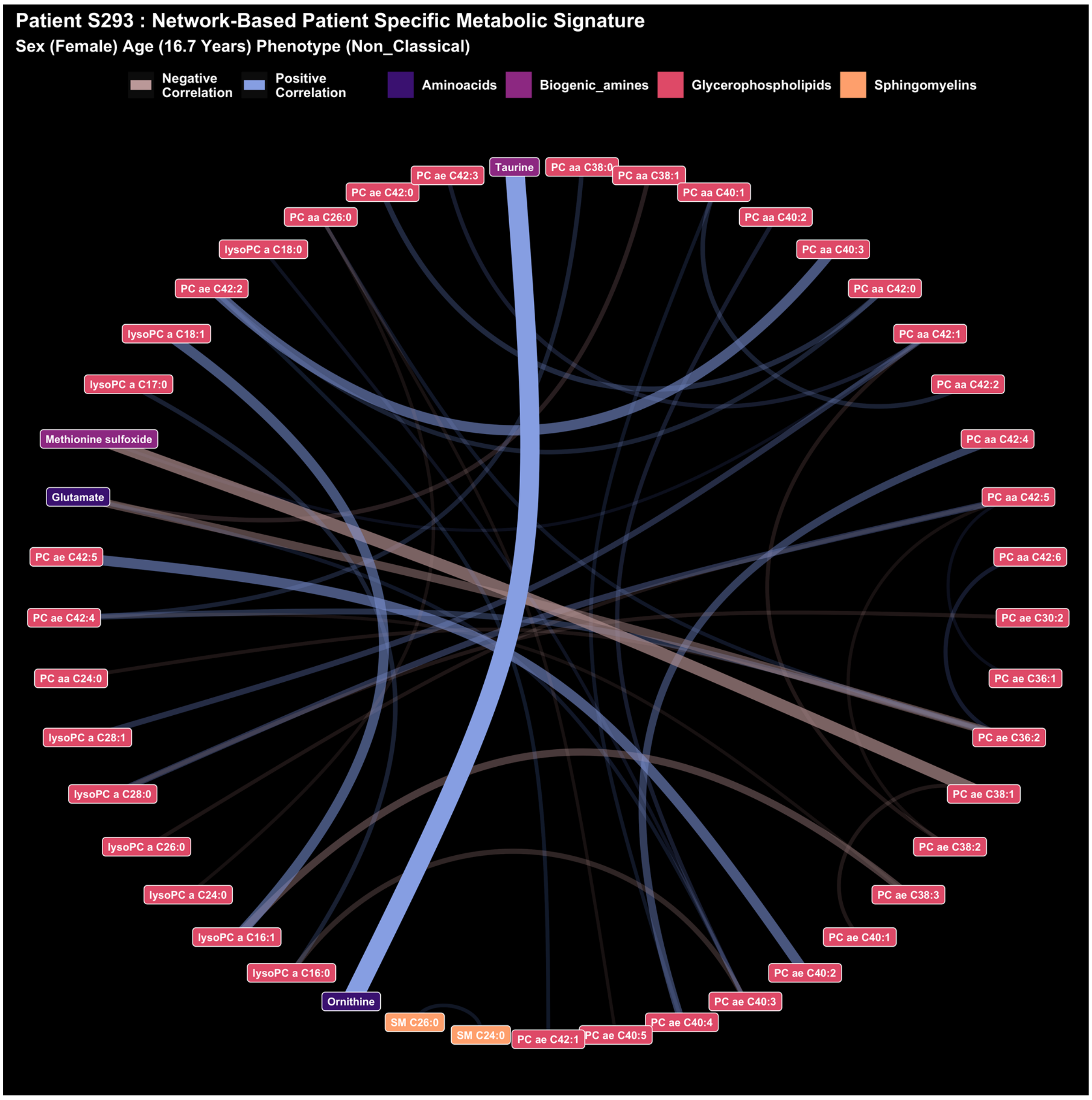

Supplementary Figure S2. Network-based patient specific metabolic signature (Patient S293).

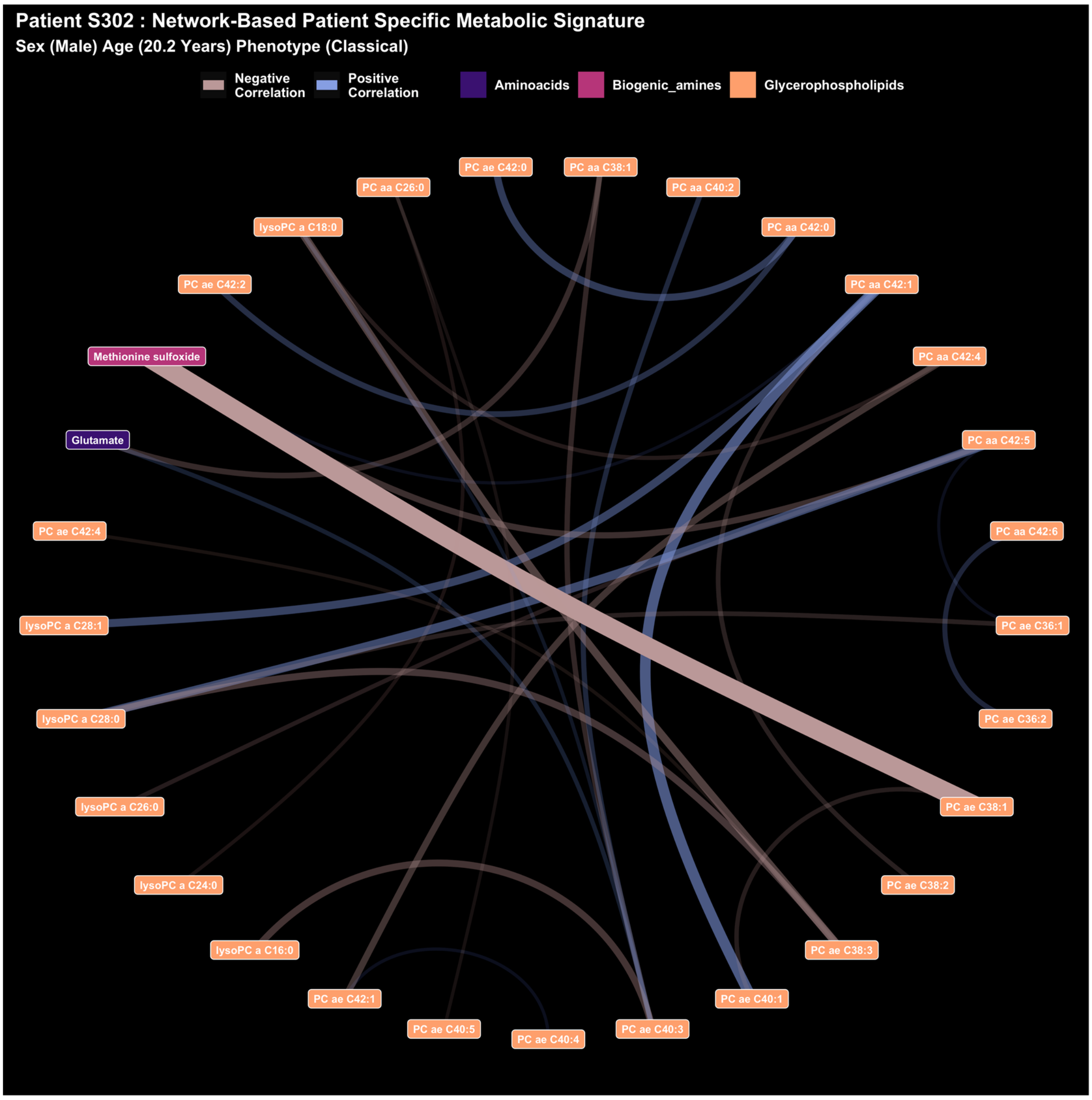

Supplementary Figure S3. Network-based patient specific metabolic signature (Patient S302).

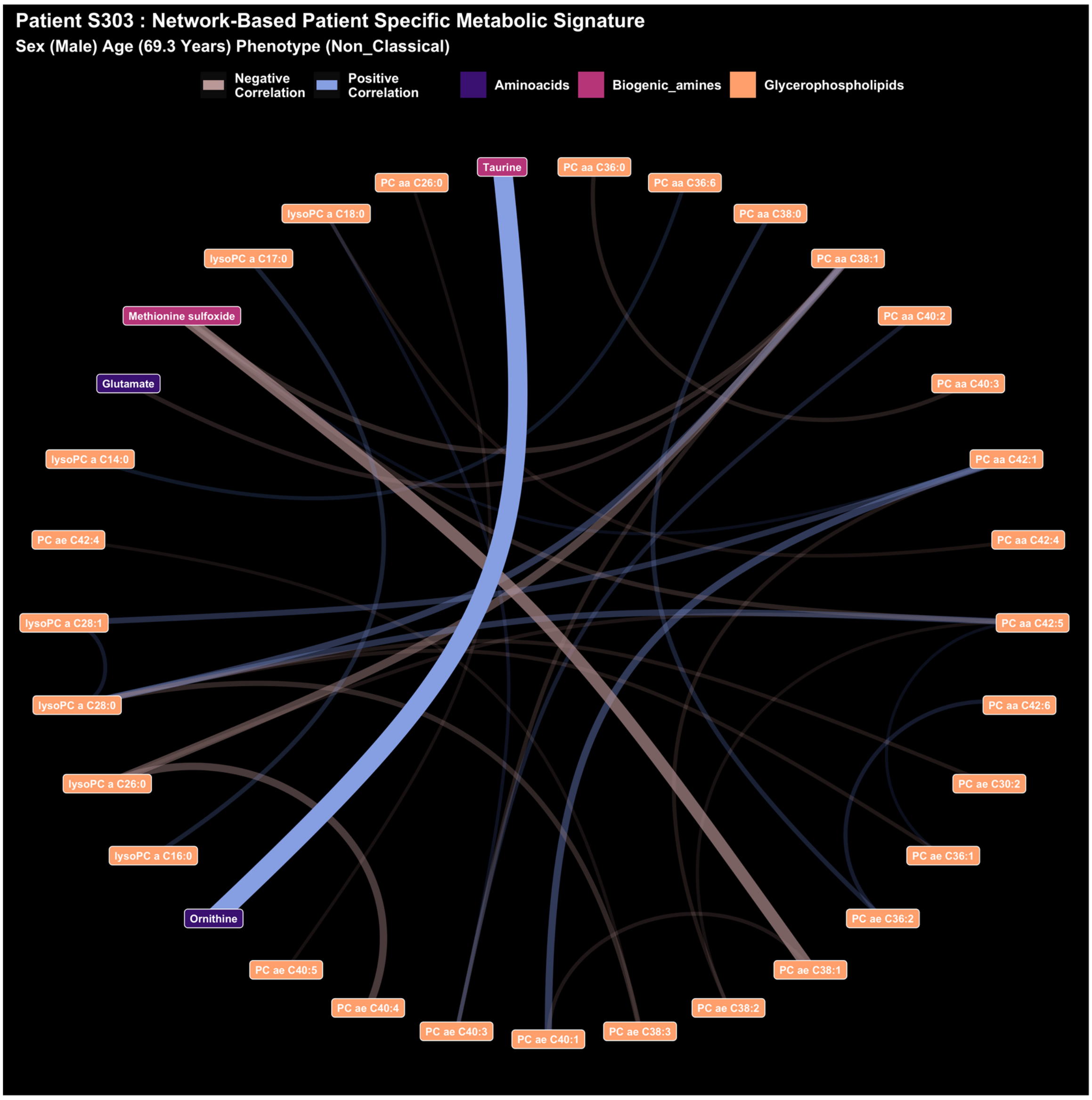

Supplementary Figure S4. Network-based patient specific metabolic signature (Patient S303).

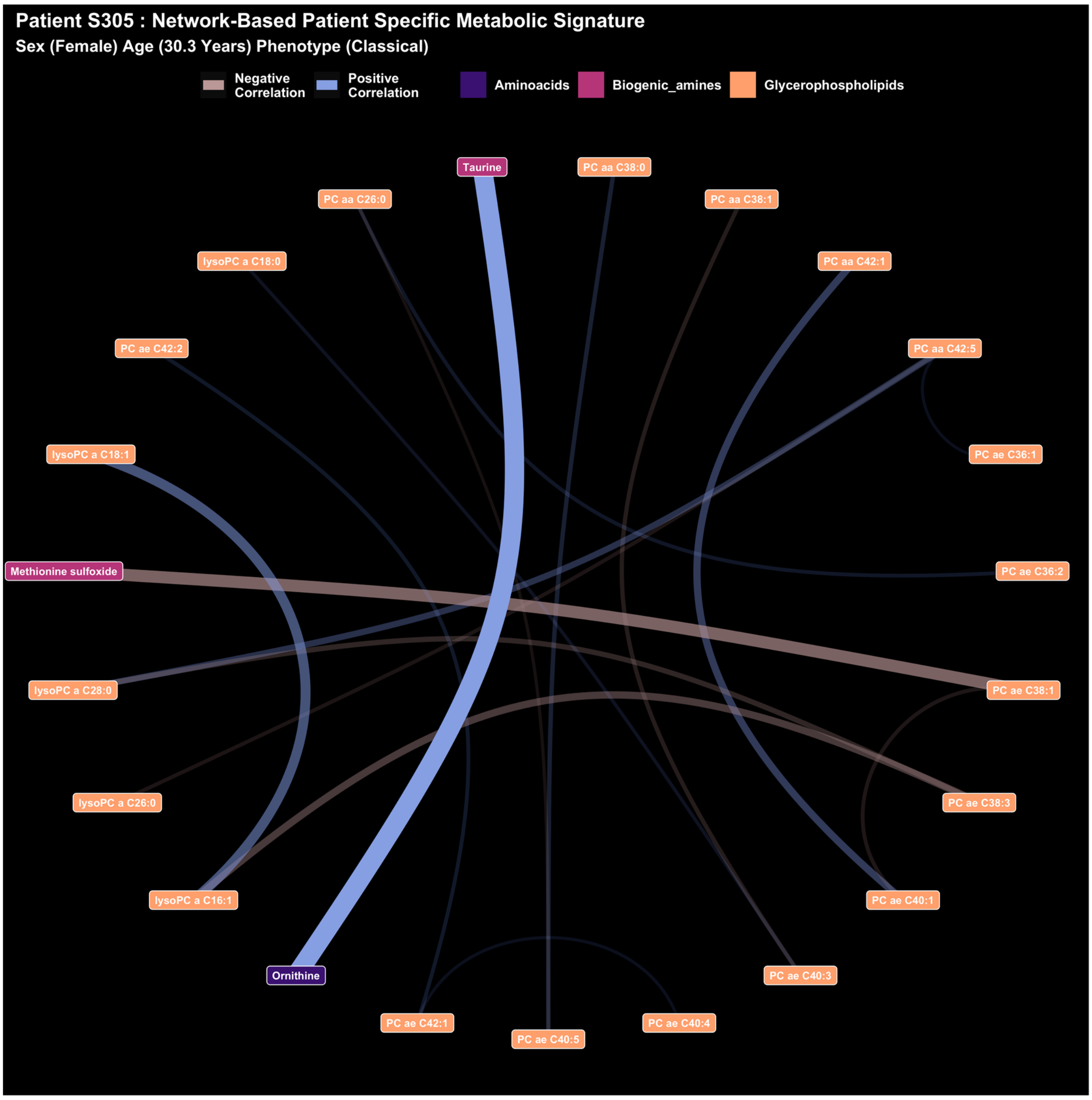

Supplementary Figure S5. Network-based patient specific metabolic signature (Patient S305).

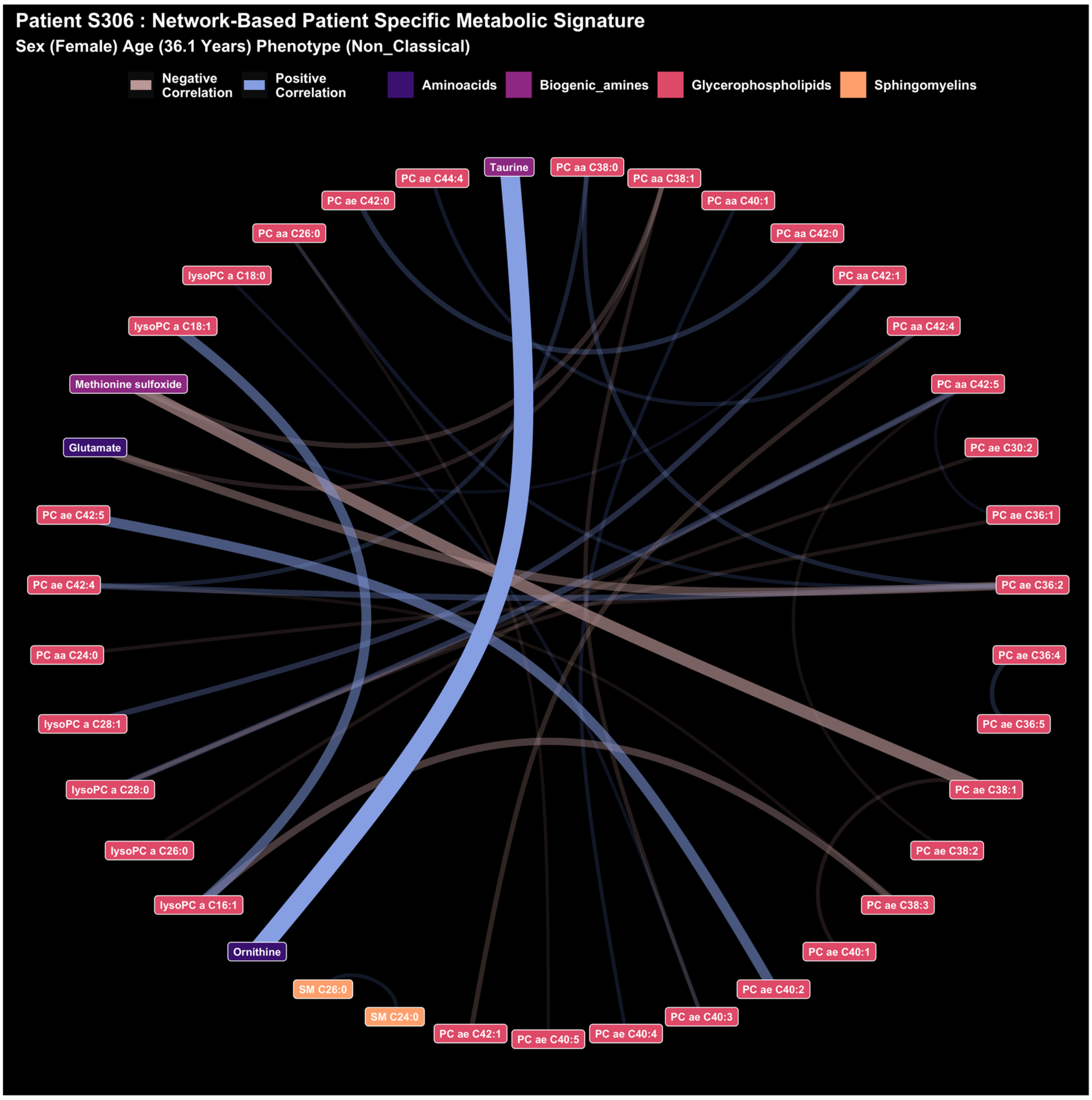

Supplementary Figure S6. Network-based patient specific metabolic signature (Patient S306).

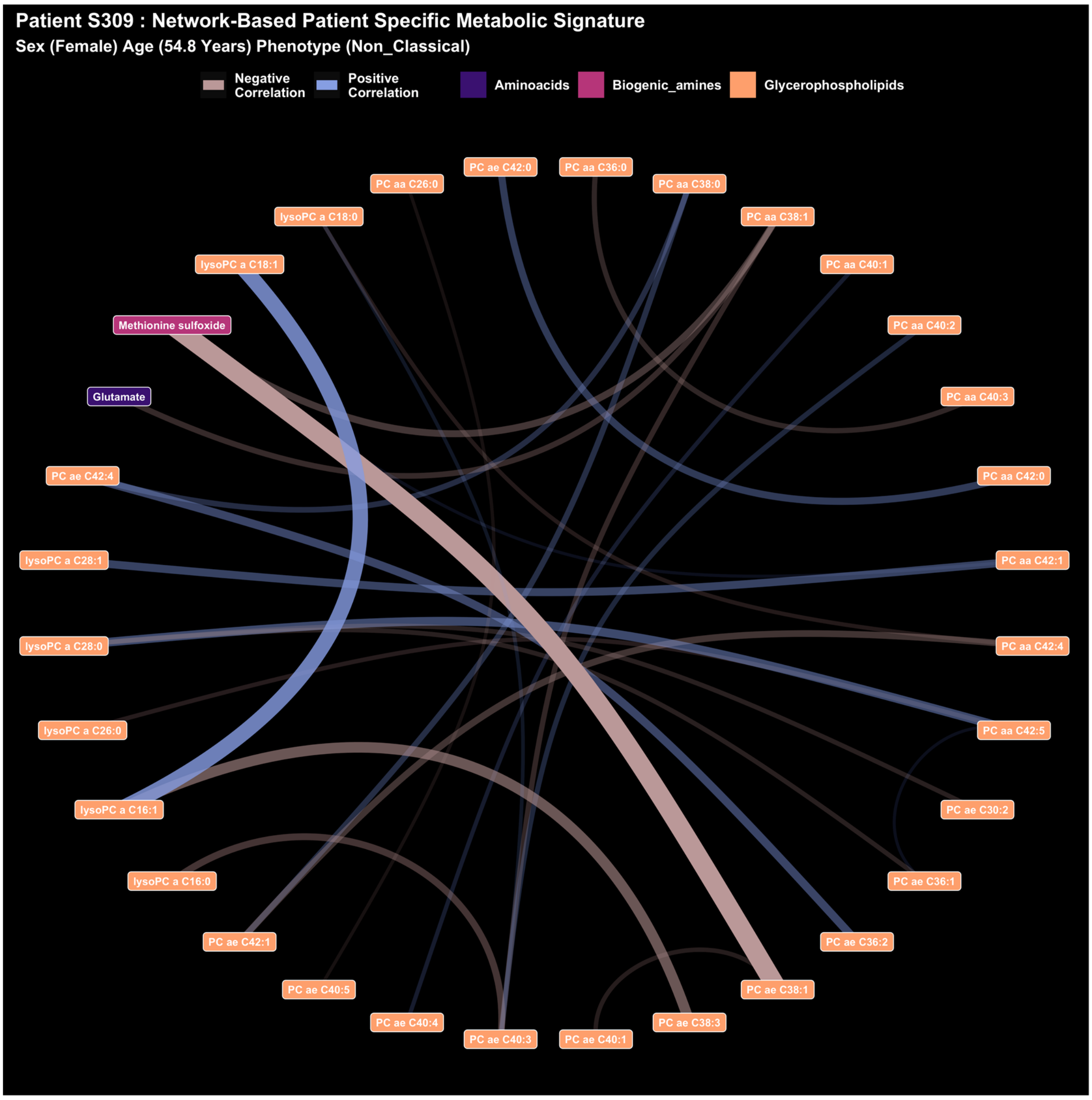

Supplementary Figure S7. Network-based patient specific metabolic signature (Patient S309).

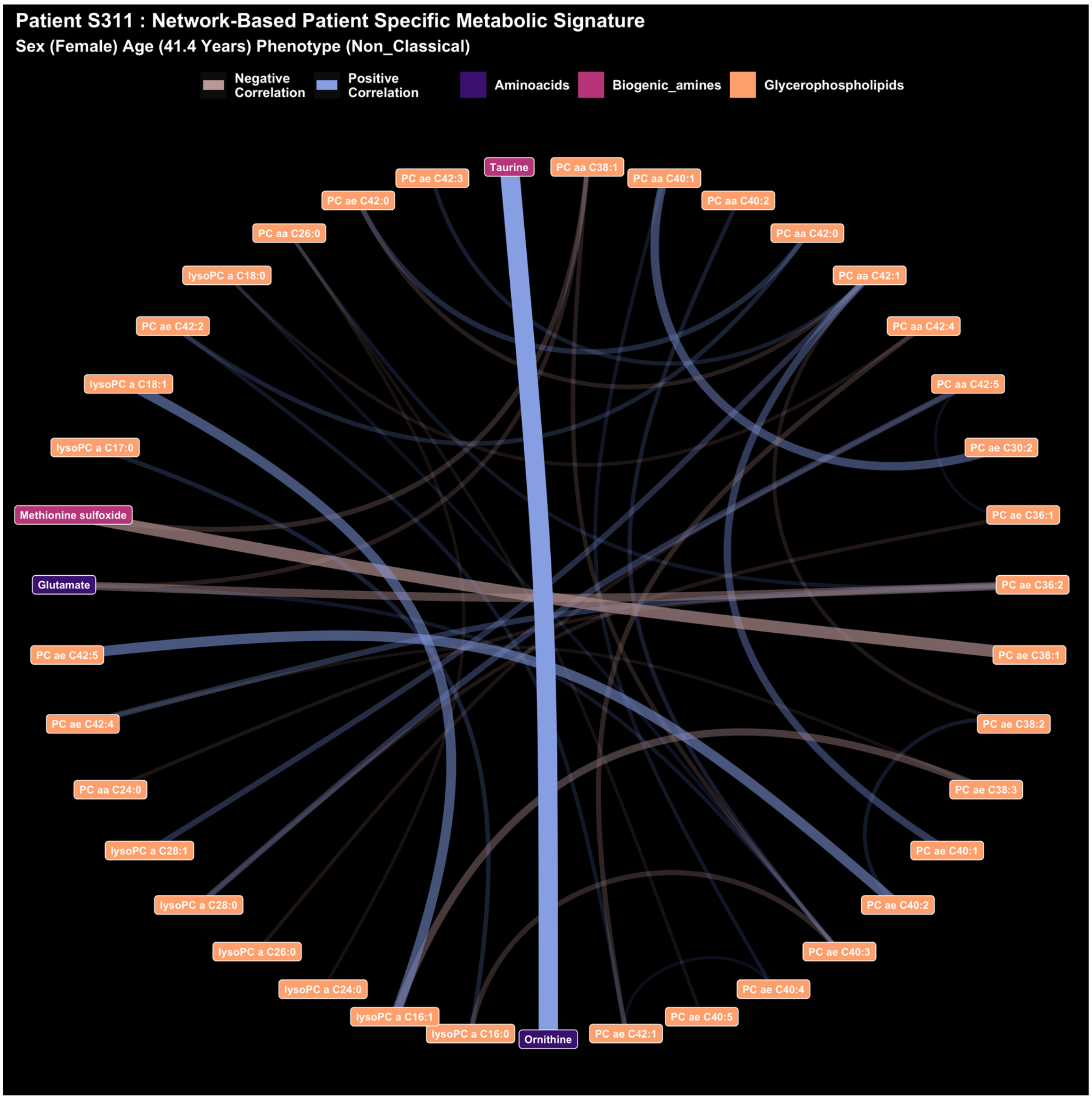

Supplementary Figure S8. Network-based patient specific metabolic signature (Patient S311).

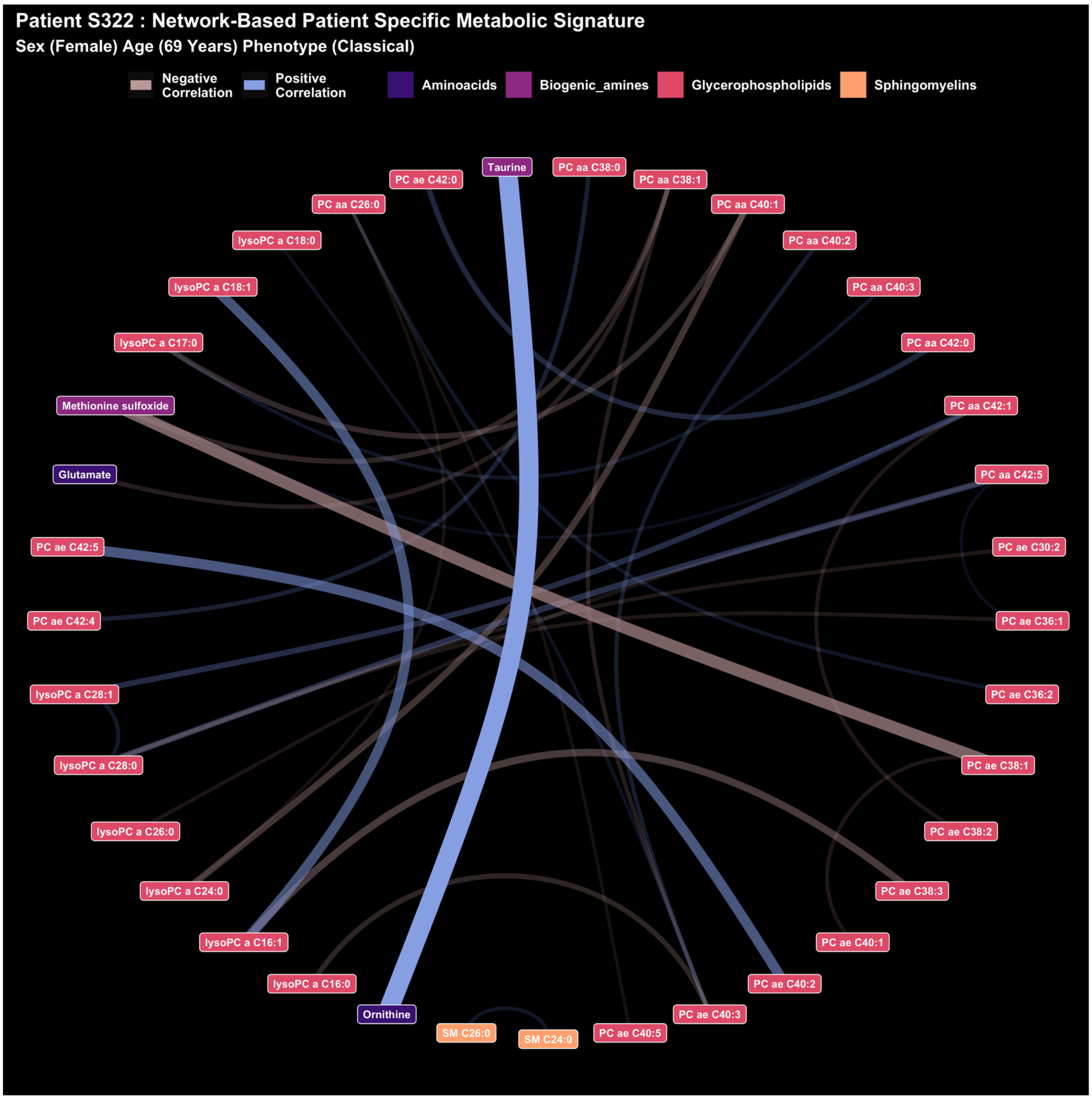

Supplementary Figure S9. Network-based patient specific metabolic signature (Patient S322).

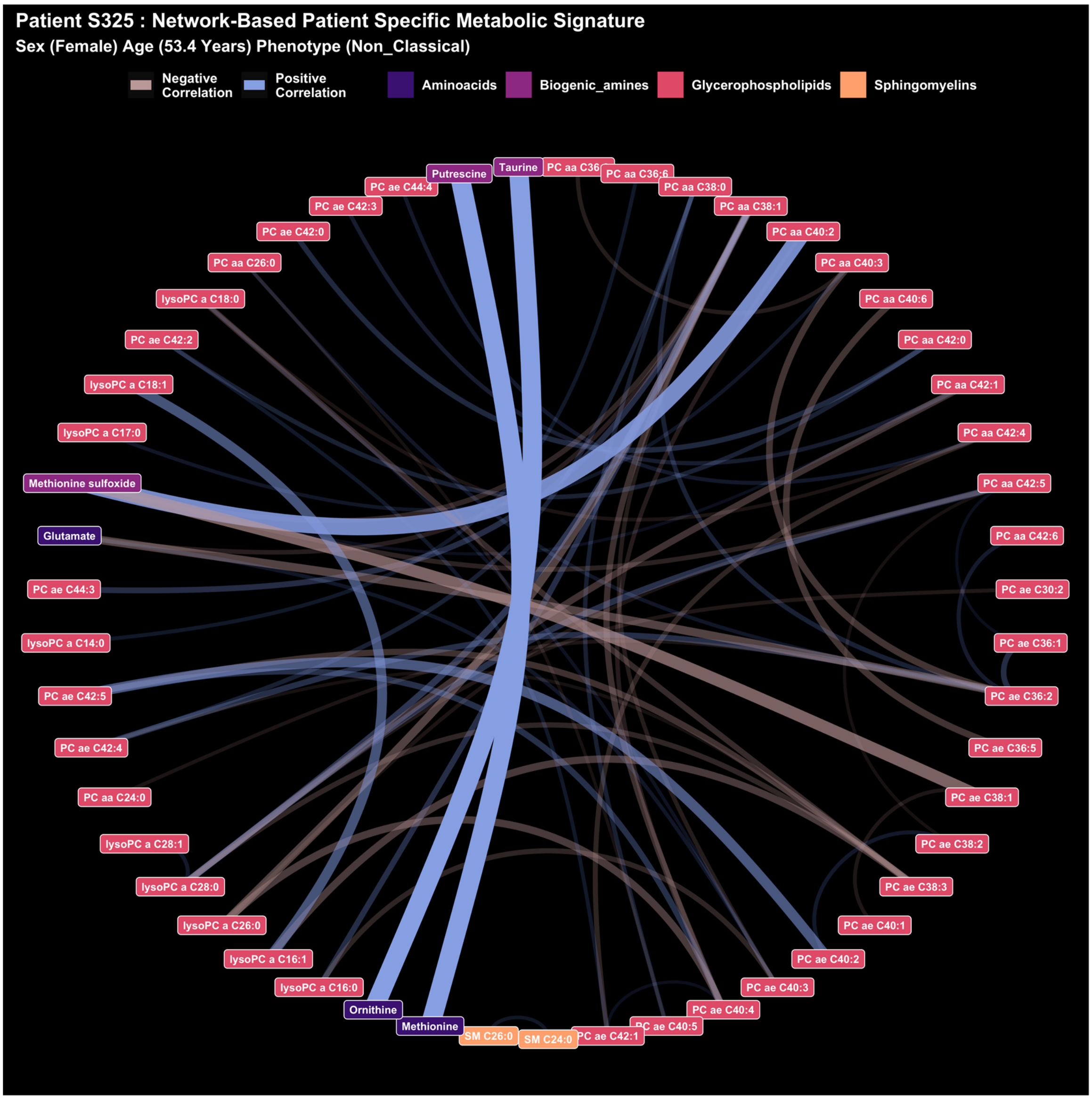

Supplementary Figure S10. Network-based patient specific metabolic signature (Patient S325).

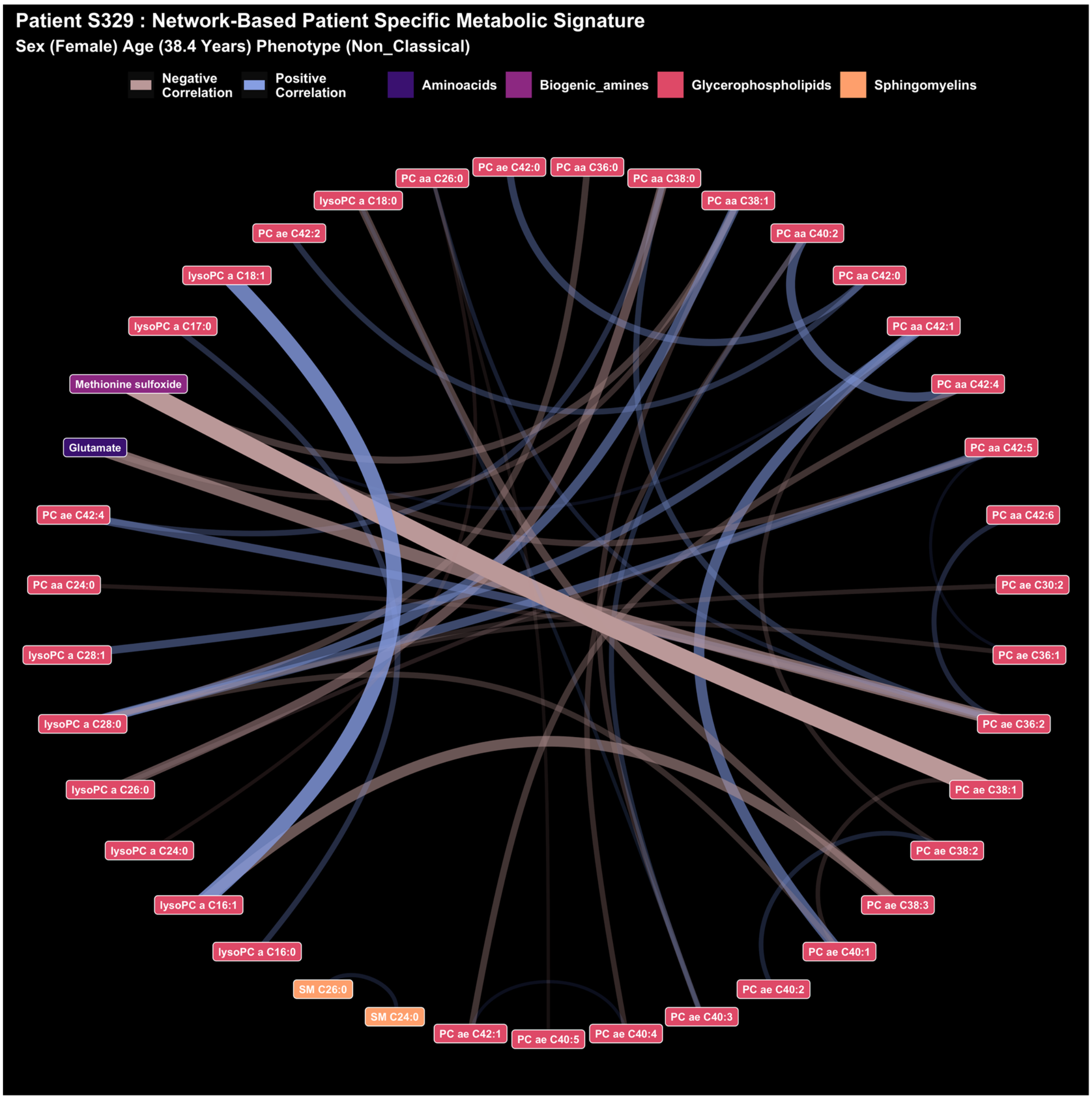

Supplementary Figure S11. Network-based patient specific metabolic signature (Patient S329).

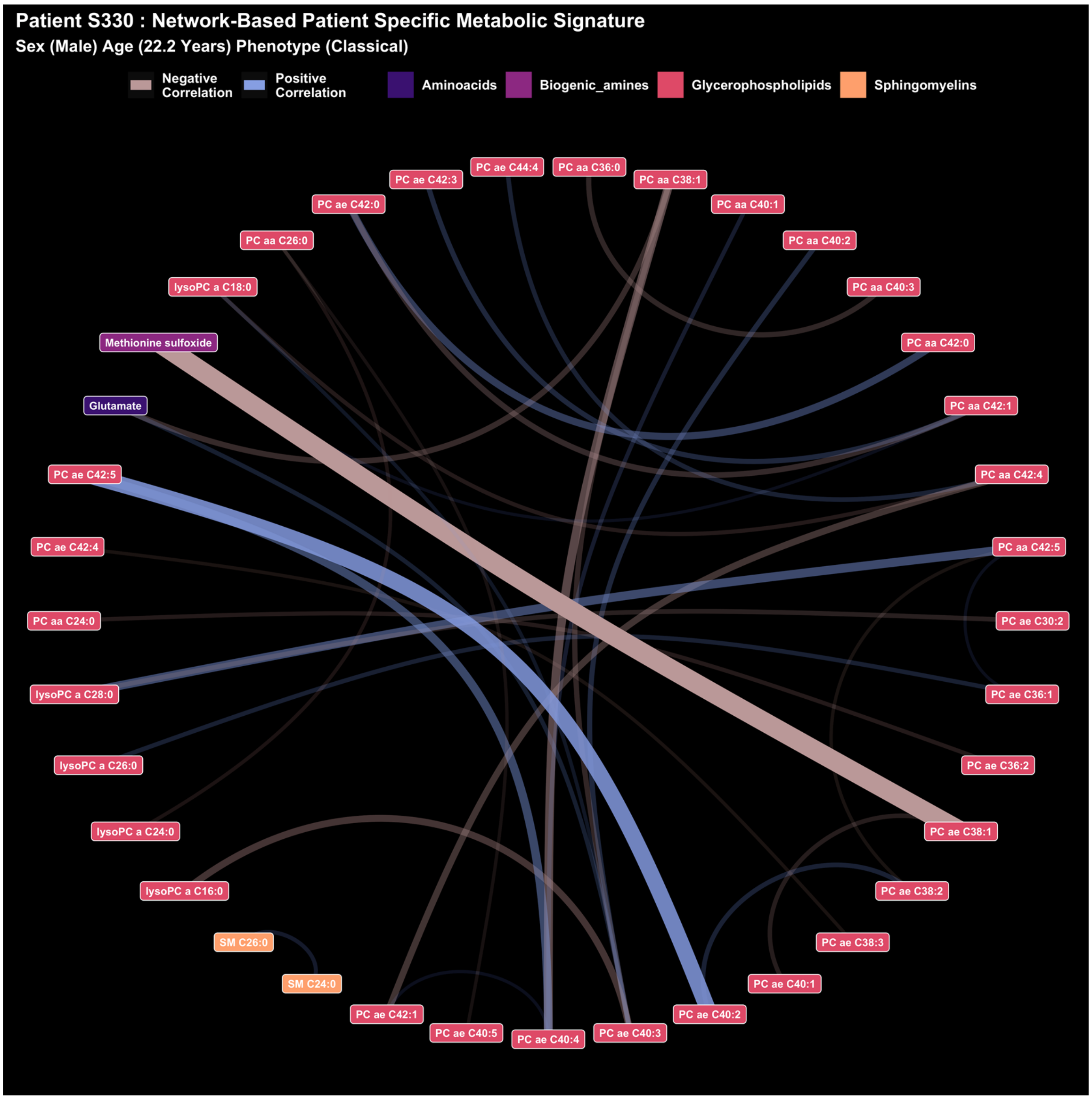

Supplementary Figure S12. Network-based patient specific metabolic signature (Patient S330).

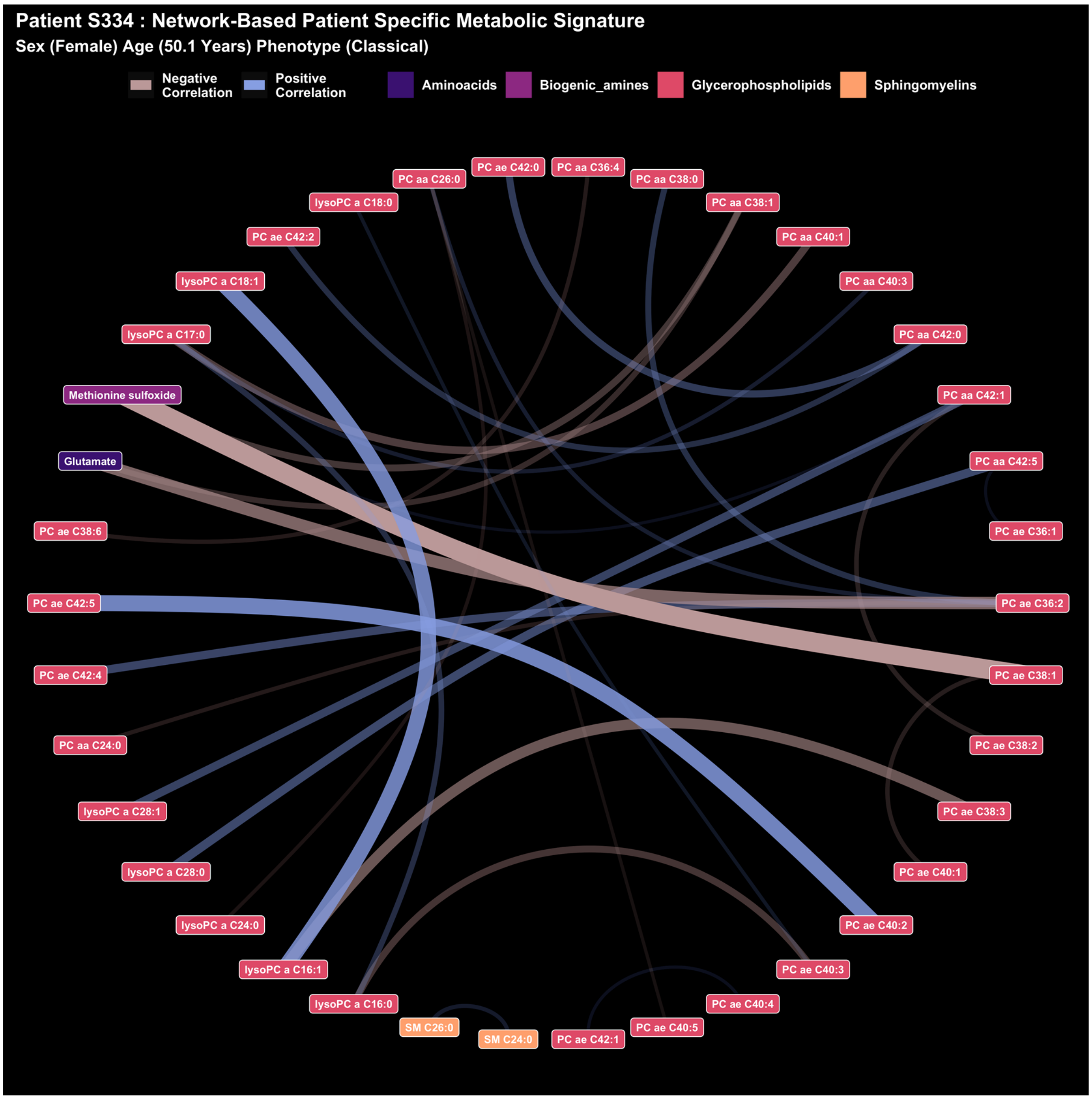

Supplementary Figure S13. Network-based patient specific metabolic signature (Patient S334).

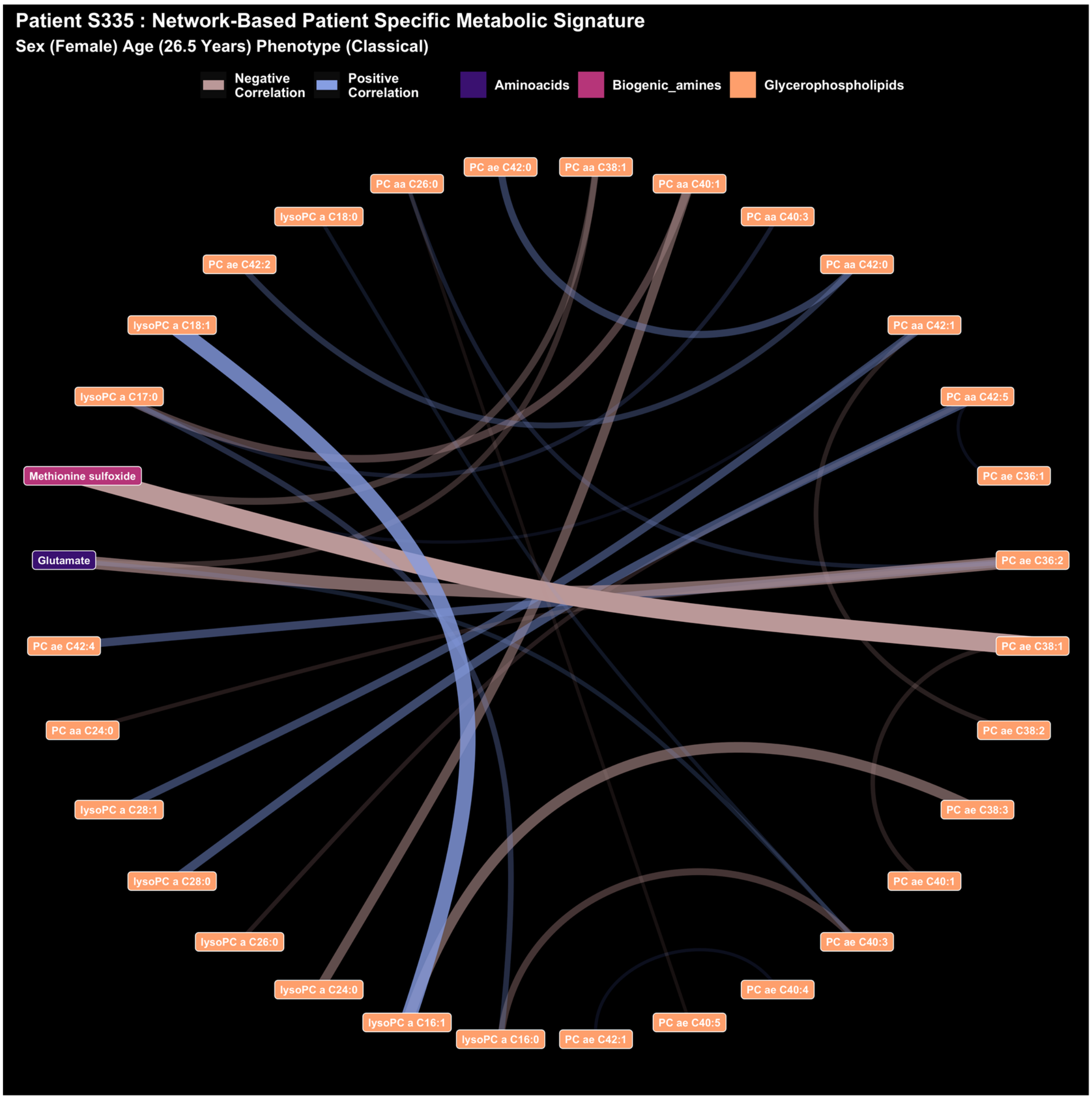

Supplementary Figure S14. Network-based patient specific metabolic signature (Patient S335).

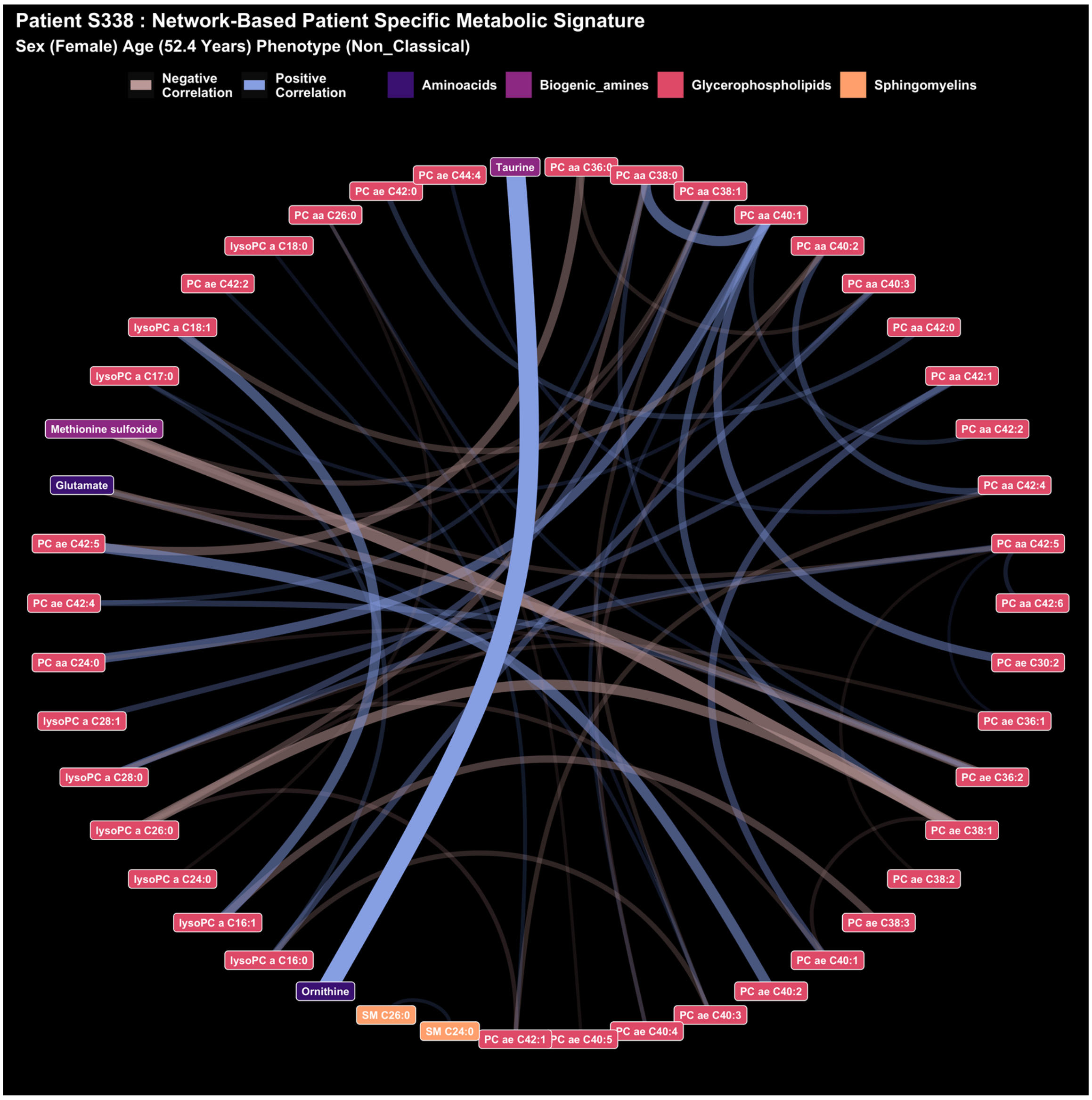

Supplementary Figure S15. Network-based patient specific metabolic signature (Patient S338).

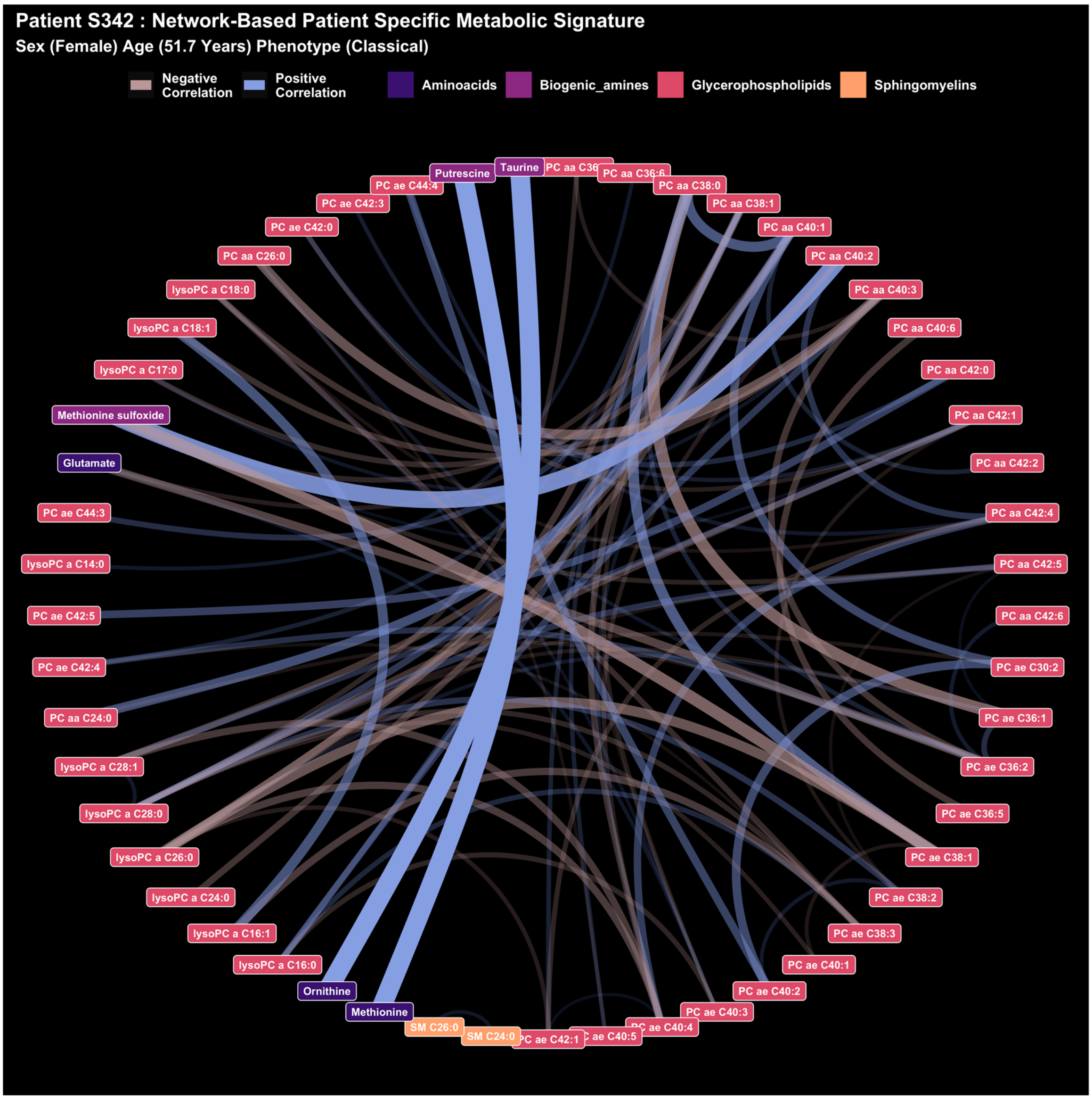

Supplementary Figure S16. Network-based patient specific metabolic signature (Patient S342).

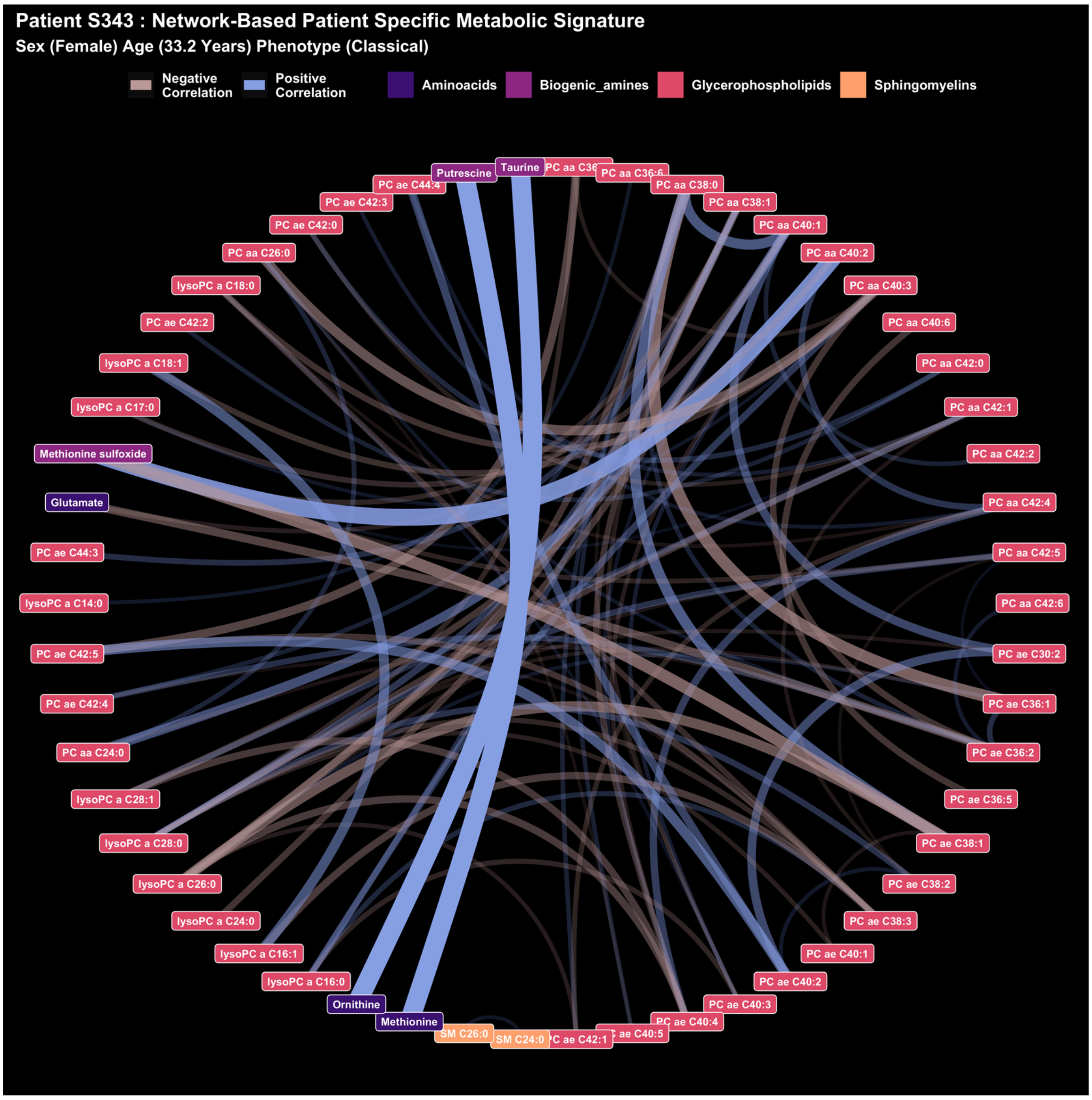

Supplementary Figure S17. Network-based patient specific metabolic signature (Patient S343).

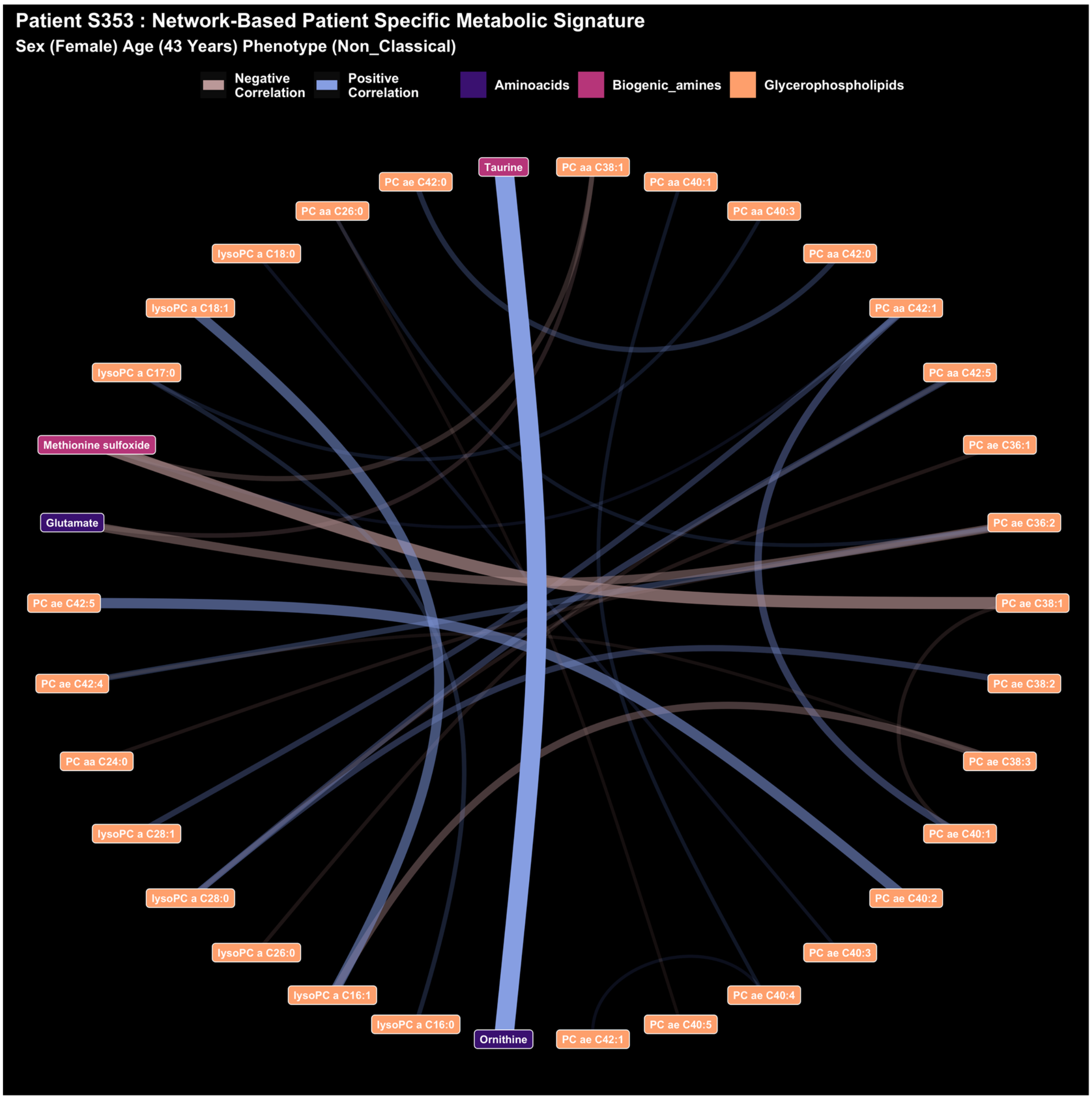

Supplementary Figure S18. Network-based patient specific metabolic signature (Patient S353).

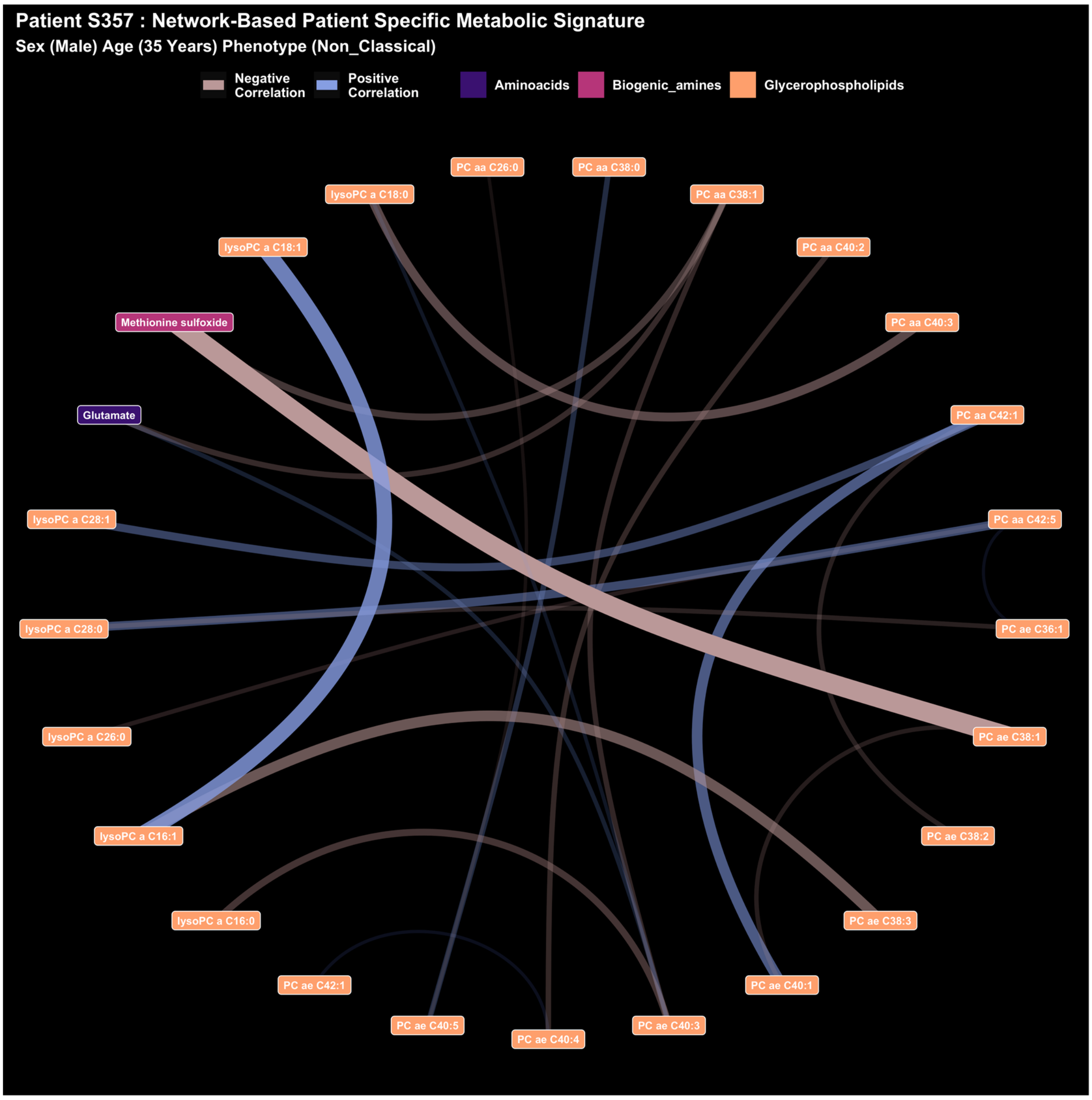

Supplementary Figure S19. Network-based patient specific metabolic signature (Patient S357).

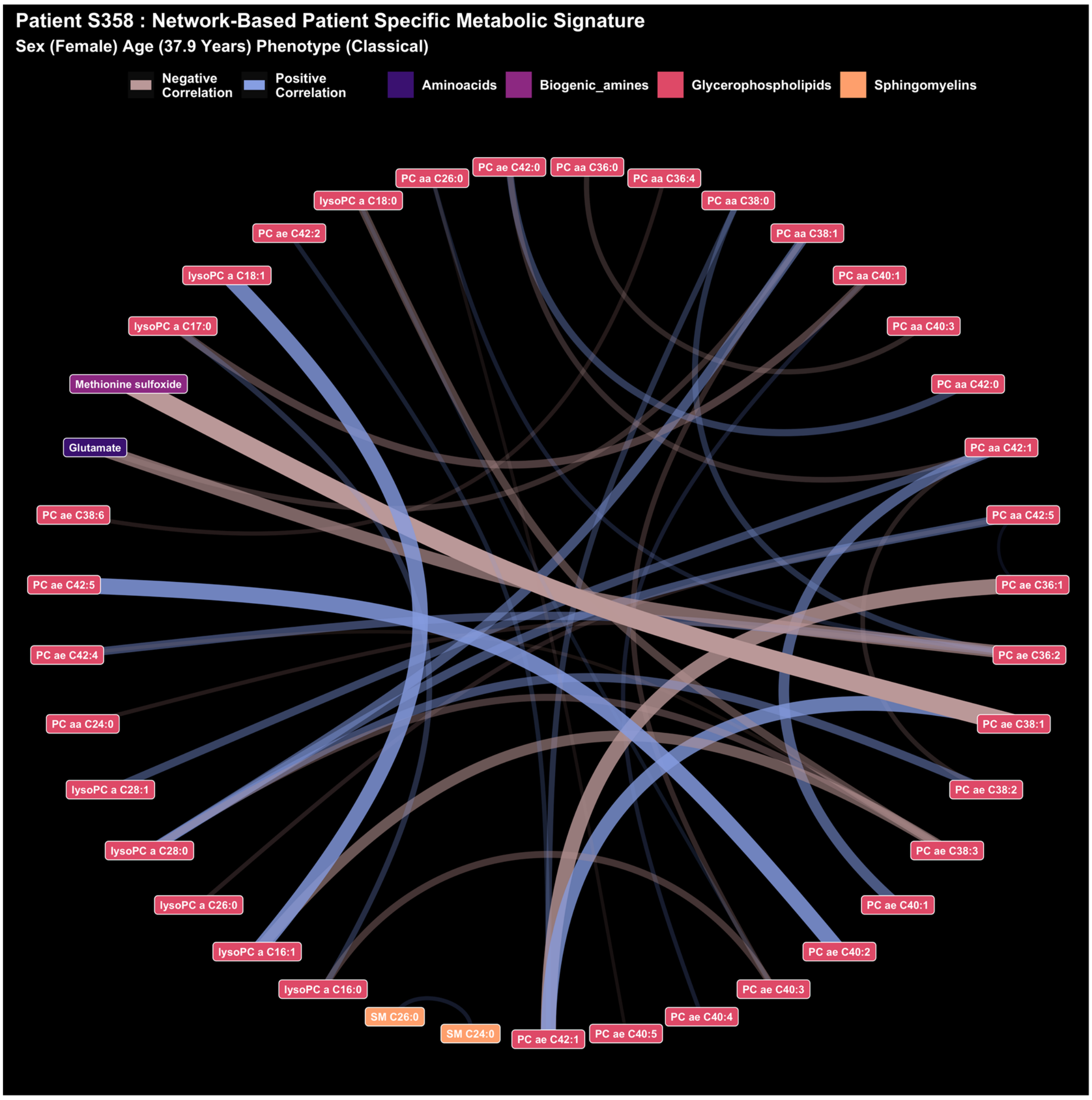

Supplementary Figure S20. Network-based patient specific metabolic signature (Patient S358).

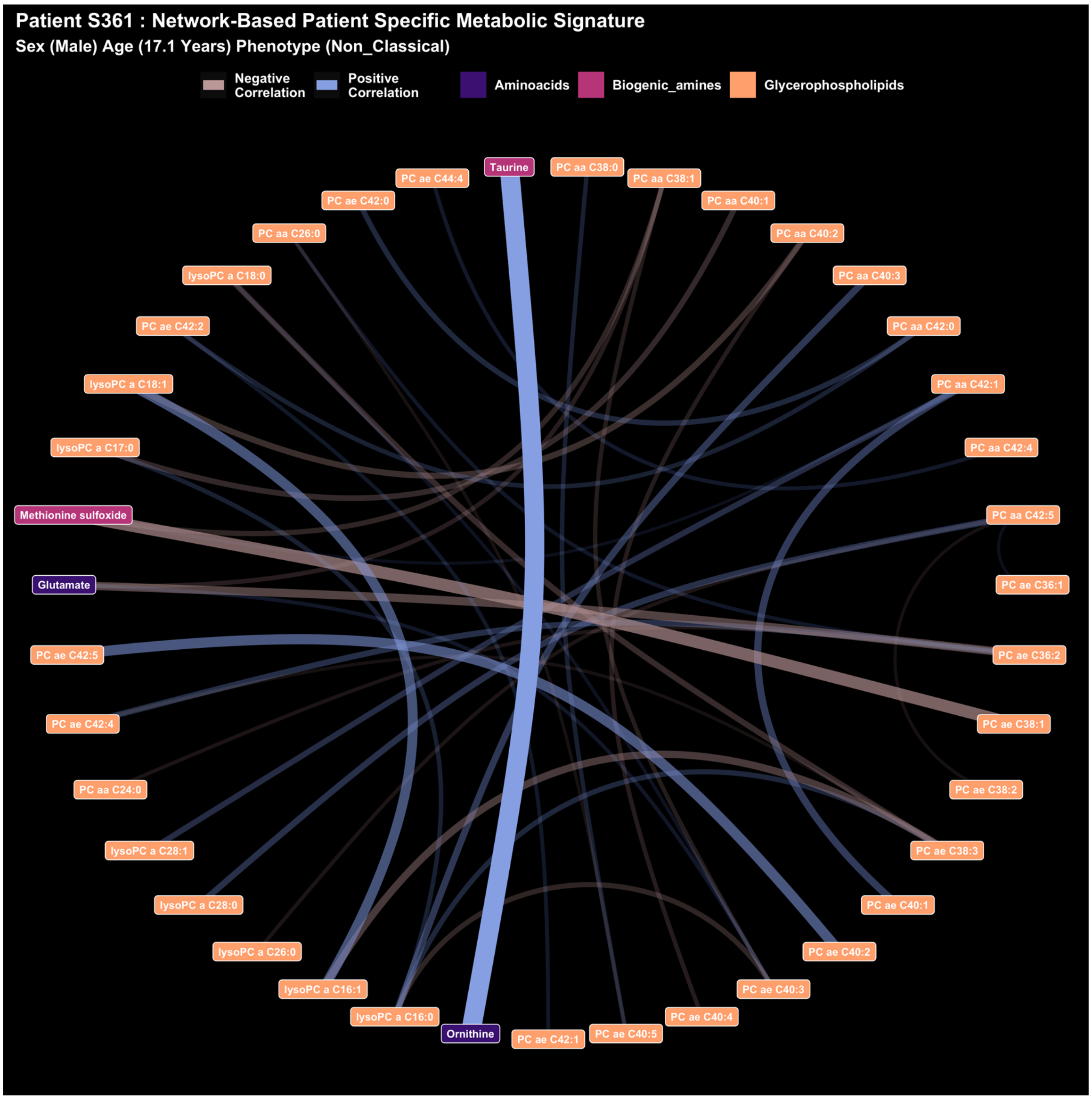

Supplementary Figure S21. Network-based patient specific metabolic signature (Patient S361).

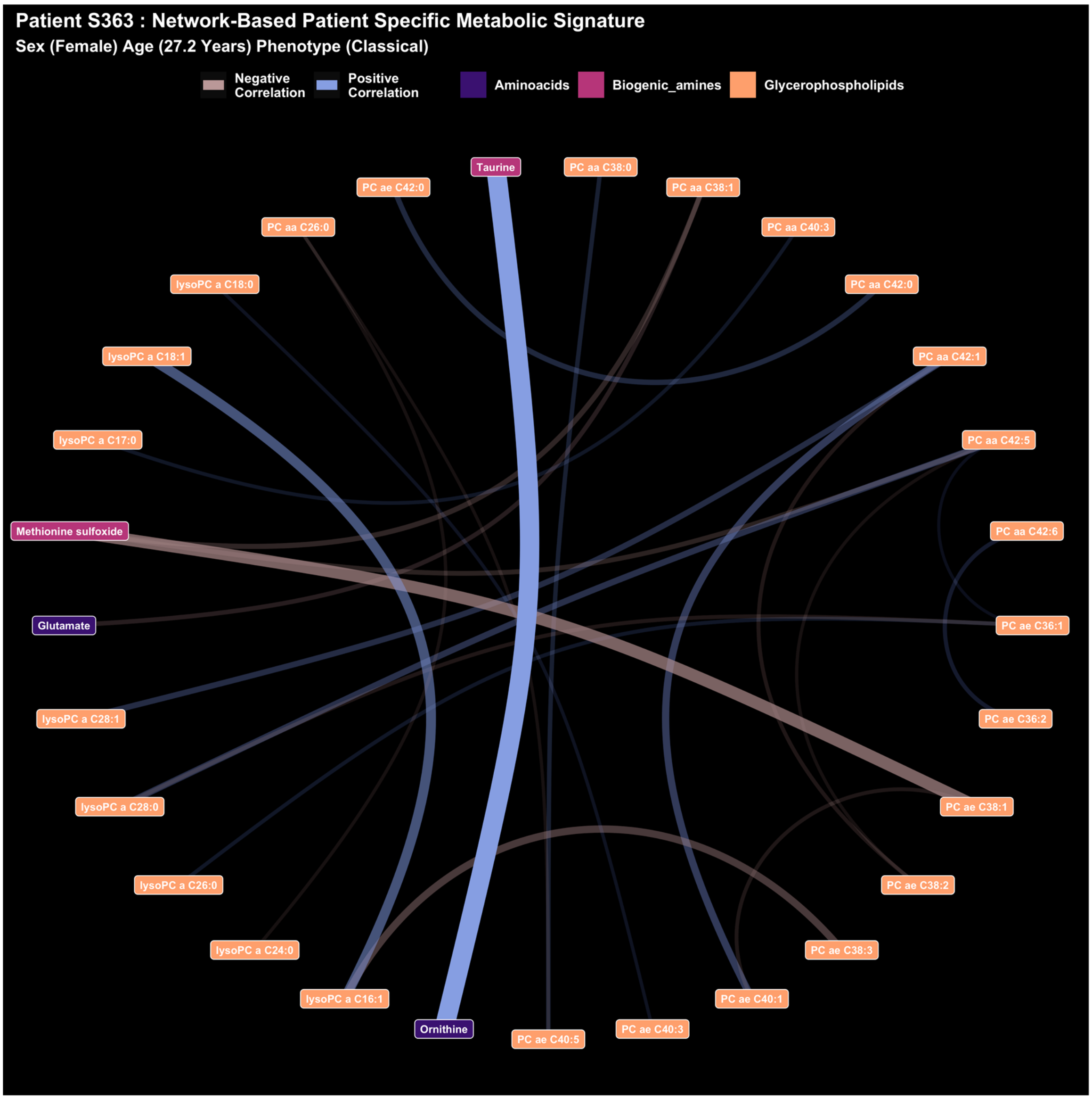

Supplementary Figure S22. Network-based patient specific metabolic signature (Patient S363).
